# Cocoa Polyphenols Alter the Fecal Microbiome Without Mitigating Colitis in Mice Fed Healthy or Western Basal Diets

**DOI:** 10.3390/nu17152482

**Published:** 2025-07-29

**Authors:** Eliza C. Stewart, Mohammed F. Almatani, Marcus Hayden, Giovanni Rompato, Jeremy Case, Samuel Rice, Korry J. Hintze, Abby D. Benninghoff

**Affiliations:** 1 Department of Animal, Dairy and Veterinary Sciences, Utah State University, 4815 Old Main Hill, Logan, UT 84322, USA; elizastewart@usu.edu (E.C.S.); mohammed.almatani@usu.edu (M.F.A.); giovanni.rompato@usu.edu (G.R.);; 2 Department of Pharmacology, College of Pharmacy, King Khalid University, Al Fara, Abha 62223, Saudi Arabia; 3 Department of Nutrition, Dietetics and Food Sciences, Utah State University, 8700 Old Main Hill, Logan, UT 84322, USA; korry.hintze@usu.edu

**Keywords:** gut microbiome, cocoa polyphenols, Western diet, colitis, inflammation, gut health, flavanols, gut barrier function, inflammatory signaling, microbial diversity

## Abstract

**Background/Objectives**: Chronic inflammation and Western-style diets elevate colorectal cancer (CRC) risk, particularly in individuals with colitis, a feature of inflammatory bowel disease (IBD). Diets rich in polyphenol-containing functional foods, such as cocoa, may reduce gut inflammation and modulate the gut microbiome. This study investigated the impact of cocoa polyphenol (CP) supplementation on inflammation and microbiome composition in mice with colitis, fed either a healthy or Western diet, before, during, and after the onset of disease. We hypothesized that CPs would attenuate inflammation and promote distinct shifts in the microbiome, especially in the context of a Western diet. **Methods**: A 2 × 2 factorial design tested the effects of the basal diet (AIN93G vs. total Western diet [TWD]) and CP supplementation (2.6% *w*/*w* CocoaVia™ Cardio Health Powder). Inflammation was induced using the AOM/DSS model of colitis. **Results**: CP supplementation did not reduce the severity of colitis, as measured by disease activity index or histopathology. CPs did not alter gene expression in healthy tissue or suppress the colitis-associated pro-inflammatory transcriptional profile in either of the two diet groups. However, fecal microbiome composition shifted significantly with CPs before colitis induction, with persistent effects on several rare taxa during colitis and recovery. **Conclusions**: CP supplementation did not mitigate inflammation or mucosal injury at the tissue level, nor did it affect the expression of immune-related genes. While CPs altered microbiome composition, most notably in healthy mice before colitis, these shifts did not correspond to changes in inflammatory signaling. Basal diet remained the primary determinant of inflammation, mucosal damage, and colitis severity in this model.

## 1. Introduction

Chronic inflammation is a well-established contributor to the development of colorectal cancer (CRC), particularly in individuals with inflammatory bowel disease (IBD), where sustained immune activation promotes tissue damage, dysregulated repair, and accumulation of oncogenic mutations as (reviewed in [1,2]). The risk of colitis-associated CRC increases with disease duration and severity of inflammation [3,4]. Mouse models of colitis and CRC demonstrate similar patterns of immune cell infiltration and inflammatory signaling observed in human disease [5,6,7].

The gut microbiome plays a central role in regulating intestinal inflammation. In healthy individuals, a diverse microbial community supports colon health by producing short-chain fatty acids, resisting pathogen colonization, and modulating immune responses [8]. Dysbiosis is characterized by reduced diversity, altered Firmicutes/Bacteroidota (F:B) ratios, and the loss of beneficial taxa, such as *Akkermansia muciniphila* and *Faecalibacterium prausnitzii,* and is commonly observed in IBD and CRC [9,10,11]. Increases in pathobionts, such as *Fusobacterium nucleatum* and *Escherichia coli*, have been linked to barrier disruption and pro-inflammatory signaling, with some species identified directly within tumor tissue [12,13].

Diet has a strong influence on both inflammatory tone and the gut microbiome [14]. Western dietary patterns, characterized by high consumption of refined carbohydrates, saturated fats, and low intake of fiber and micronutrients, promote chronic low-grade inflammation and dysbiosis [15]. The total Western diet (TWD) is a rodent model that replicates the nutrient profile of the average American diet, and TWD-fed mice exhibit increased susceptibility to colitis and CRC in chemically induced models [5,16,17].

Dietary polyphenols are bioactive compounds found in plant-based foods that have attracted attention for their anti-inflammatory and microbiome-modulating properties. Cocoa (*Theobroma cacao*) is particularly rich in flavanols, such as catechins, epicatechins, and procyanidins [18]. Although polyphenol absorption in the small intestine is limited, unabsorbed compounds reach the colon, where gut microbes metabolize them into bioactive derivatives [19]. In vitro and animal studies suggest that cocoa polyphenols reduce inflammatory signaling and improve histological markers of colitis [20,21,22]. Cocoa intake has also been shown to enrich beneficial bacterial taxa, including *Bifidobacterium* and *Lactobacillus*, in both preclinical and human studies [23,24].

Despite these promising findings, little is known about the efficacy of cocoa polyphenols in the context of a Western diet, where pro-inflammatory conditions may limit their protective potential. Previous studies have mainly tested cocoa supplementation in healthy dietary backgrounds. Whether cocoa can modify inflammation or microbiome composition in a pro-inflammatory diet setting remains unclear. This study aimed to evaluate the effects of cocoa polyphenol (CP) supplementation on colon inflammation and gut microbiome composition before, during, and after chemically induced colitis in mice fed either a healthy control diet (AIN93G) or the total Western diet (TWD). We hypothesized that CP supplementation would attenuate colitis symptoms and modulate microbiome profiles, particularly in TWD-fed mice, by reducing inflammation and promoting beneficial shifts in microbial composition.

## 2. Materials and Methods

### 2.1. Chemicals and Reagents

Azoxymethane (AOM) was purchased from Sigma-Aldrich (St. Louis, MO, USA; CAS No. 25843-45-2). Dextran sodium sulfate (DSS; reagent grade at mol. wt. ~40 kDa) was purchased from Thermo Fisher Scientific (Waltham, MA, USA). All other chemicals were purchased at reagent grade from general laboratory supply companies.

### 2.2. Animals and Experimental Diets

The Utah State University Institutional Animal Care and Use Committee approved all the animal handling and treatment procedures used in this study (IACUC #12860). Male C57BL/6J mice were obtained from Jackson Laboratories (Bar Harbor, ME, USA) at five weeks of age (total *n* = 240). Mice were kept in a pathogen-free vivarium at 18–23 °C, under 12:12 h dark/light cycle, and at a constant humidity of 50%. HEPA-filtered cages were used for housing in an IVC Air Handling Solution ventilated housing system (Tecniplast, Buguggiate, Italy) with Bed-o’ Cobs^®^ ¼ bedding (Andersons, Cincinnati, OH, USA). Nesting material was provided as enrichment to reduce stress. After a week of quarantine, mice were randomized into experimental groups using a random block design to standardize the starting body weight across the experimental groups. Mice were housed two in a cage and were ear-punched for identification. Cages were changed weekly, fresh food was provided twice a week, and autoclaved water was provided ad libitum throughout the study. Cages were identified by experimental group and sample time point to facilitate accurate administration of the experimental diets on the defined schedule.

Experimental diets were prepared by Envigo (Hackensack, NJ, USA). Diets were obtained in two lots, mixed to prevent lot effects, and then stored at 4 °C for the remainder of the study. AIN93G (AIN, cat. no. TD.160421), a diet formulated to promote rodent health and containing 3.8 kcal/g, was used as a negative control diet. The total Western diet (TWD, cat. no. TD.160422), which contains 4.4 kcal/g and reflects the macro- and micro-nutrient intake of the average American on an energy density basis, was used as a positive control [25]. CocoaVia™ Cardio Health Powder was obtained from Mars, Incorporated (McLean, VA, USA) and incorporated in either AIN or TWD basal diets at 2.6% (*w*/*w*) to achieve a polyphenol concentration of 0.2% (*w*/*w*). Adjustments were made to the carbohydrate and protein amounts in the basal diets to maintain approximately the same total carbohydrate and protein amounts as the control diets (Table S1). Given an average daily food intake of 3.5 g for a 20 g mouse, this amount of supplementation will result in approximately 5.47 mg of cocoa flavanols per mouse per day. Using a nutrient density scaling approach, this exposure translates to approximately 1525 mg of flavanols per day in humans, which can be achieved by consuming three servings of CocoaVia™ Cardio Health Powder per day. While this intake exceeds typical dietary levels for most populations, it is consistent with our previous work in this model [26] and may reflect plausible exposure levels for individuals using polyphenol supplements therapeutically, such as patients with IBD.

### 2.3. Study Design, Colitis Symptoms, and Assessment of Colon Tissue Histopathology

A 2 × 2 factorial design was used with the two main factors *basal diet* and *cocoa supplementation*, and the following four experimental groups: (1) AIN control (AIN/CON), (2) AIN + 2.6% CocoaVia™ powder (AIN/CP), (3) TWD control (TWD/CON), and (4) TWD + 2.6% CocoaVia™ powder (TWD/CP). Mice were quarantined, randomized, and housed as described above (*n* = 60 per diet group, with 20 mice per time point). Each diet group was given their basal diet from days 0 to 6, with groups AIN/CP and TWD/CP starting CP-supplemented diets on day 7 and remaining on those diets for the remainder of the study. On day 22, mice were administered AOM (10 mg/kg) intraperitoneally in sterile PBS and received 1% (*w*/*v*) DSS in their drinking water for the following 10 days. Disease activity index (DAI) scores were recorded on days 32 and 46, as previously described [5]; the observer was blinded to the experimental group assignments of the individual mice.

Body composition was determined on day 45 using MRI (EchoMRI-700; EchoMRI, Houston, TX, USA). Subsets of mice (*n* = 20 per group) were randomly selected and euthanized by CO_2_ asphyxiation at three time points throughout the study: day 21 (pre-DSS), day 33 (colitis), and day 47 (recovery). Subsets of colons from each group were collected for histopathological assessment of inflammation and mucosal injury (*n* = 12) or collection of colon mucosa (*n* = 8) by a board-certified veterinary pathologist, as previously described [5], who was blinded to the sample’s experimental group assignment. Final sample sizes are reported in Table S2.

A power analysis was conducted a priori for the primary endpoint DAI using a one-way ANOVA followed by Tukey’s Honestly Significant Difference (HSD) post hoc test, using simulated data to model a 4-group design (*n* = 20 per group, total *n* = 80), accounting for all combinations of diet × supplement at each time point, with a pooled standard deviation of 2.14 units. Assuming a 30% difference between group means (Cohen’s d = 0.92), the analysis estimated a statistical power of 81.2% (α = 0.05) to detect significant pairwise differences while controlling for multiple comparisons using Tukey’s HSD test.

### 2.4. Nanostring Gene Expression

Gene expression profiling of colon mucosa samples was performed using the NanoString nCounter platform and analyzed with nSolver software (version 4.0.7), as previously described in detail [5,27]. Samples were blinded to the experimental group before processing, with only the project director (A.D.B.) and laboratory manager (G.R.) having access to the sample identification code and experimental group key. Samples remained blinded throughout preparation and processing. Total RNA was extracted from the colon mucosa using TriReagent (Sigma Aldrich, St. Louis, MO, USA) according to the manufacturer’s instructions. The resultant RNA was further purified, and genomic DNA was removed using the RNeasy Mini Kit with DNase treatment (Qiagen, Valencia, CA, USA). RNA was dissolved in nuclease-free water and quantified using Qubit (Thermo Fisher), and RNA quality was verified by Agilent 2200 TapeStation analysis (Agilent, Santa Clara, CA, USA). A limited number of samples per subgroup (*n* = 3–6) passed RNA quality control parameters with sufficiently high concentration for further analysis (Table S2).

Expression analysis was conducted using the nCounter Mouse PanCancer Immune Profiling Panel (NanoString, Bothell, WA, USA), which targets 750 immune-related genes and includes built-in controls. One sample was excluded because it failed the quality control parameters. Following hybridization and processing on the nCounter MAX system (NanoString), the raw data were background-corrected using internal negative controls and normalized using positive controls. The geNorm-identified housekeeping genes were then used for normalization. Transcript count ratios were calculated relative to the AIN/CON diet group at the pre-DSS time point, serving as the reference dataset, and then log_2_-transformed. Differential expression analysis was conducted using the nSolver and the Advanced Analysis Module (NanoString), which applies appropriate regression models and adjusts *p*-values using the Benjamini–Hochberg (BH) false discovery rate correction to generate adjusted *q*-values. A significant difference in gene expression was defined as a fold change > 1.5 with the BH-adjusted *q*-value < 0.05. Pathway-level analysis included directed gene set analysis (GSA) significance scores, as well as pathway Z scores derived from principal component analysis, as described in depth previously [5,27]. Hierarchical clustering analyses were performed on log_2_-transformed data using Euclidean distance with average linkage, as implemented in ClustVis [28].

### 2.5. Microbiome Profiling by 16S rRNA Sequencing

The complete procedure for sample processing and sequencing is found in Rodriguez et al. [16], with only a few modifications. Samples were blinded to the experimental group before processing, with only the project director (A.D.B.) and student researcher (E.C.S.) having access to the sample identification code and experimental group key. Samples remained blinded throughout the preparation, sequencing, and data processing stages.

Briefly, fecal samples were collected at three time points: pre-DSS, colitis, and recovery. Fecal samples were stored at −20 °C until DNA extraction, which was performed using the QIAamp DNA Stool Mini Kit (Qiagen, Fredrick, MD, USA), followed by determination of DNA concentration and quality using UV spectrophotometry (Nanodrop 2000, Thermo Fisher, Waltham, MA, USA). Samples were then diluted to 5 ng/µL in tris-EDTA buffer (TE, pH 8.0). The V4 region of the 16S rRNA gene was amplified using primers 515F and 806R [29] and a two-step PCR protocol with Platinum HS reagents (Thermo Fisher Scientific, Waltham, MA, USA). Amplicon size (~254 bp) was verified by gel electrophoresis. PCR products were purified using AMPure XP beads (Beckman Coulter, Indianapolis, IN, USA), quantified using the Quant-IT Picogreen dsDNA Assay (Thermo Fisher Scientific), and normalized to 1 ng/μL. Samples were pooled and stored at −20 °C prior to sequencing. Paired-end sequencing (2 × 250 bp) was performed using the Illumina MiSeq platform with the MiSeq Reagent Kit v2 (Illumina, San Diego, CA, USA). Final sample sizes are reported in Table S2.

Microbiota sequences were processed using QIIME 2 [30] and DADA2 [31] to filter for quality and length, remove chimeras, and generate a table of amplicon sequence variants (ASVs). To assign taxonomy, the Qiime feature-classifier classify-sklearn command was used with a classifier pre-trained for the V4 region, silva-138-99-515-806-nbclassifier.qza, and the Silva database (138 SSU) [32].

### 2.6. Microbiome Sequencing Data Analysis

Diversity and abundance analyses were performed using the Microbiome Analyst Marker Data Profiling module [33]. The ASV table was further processed to remove samples with low counts (minimum four counts with 20% prevalence in samples) and low variance (10% removed based on interquartile range). The resulting filtered ASV table was then rarefied to the minimum library size and normalized by total sum scaling. Alpha diversity was measured as the observed number of ASVs (number sequenced), the Chao 1 richness (number of species represented), and the Shannon index (a weighted abundance measure of species present). Alpha diversity scores were analyzed for the entire dataset to identify potential outlier microbiome profiles by applying the robust outlier test (ROUT) with a conservative *Q* value of 0.1% (GraphPad Prism v. 10.0.1, San Diego, CA, USA), meaning that there is a ≤0.1% chance of excluding a data point as an outlier in error; this approach was determined a priori. No outlier samples were identified.

Beta diversity was calculated using unweighted UniFrac distances (sensitive to rare taxa) and weighted UniFrac distances (weighted by abundance). Both measures were reported as principal coordinate plots (PCoAs), using the first two coordinates, with *R*^2^ > 0.10 and a PERMANOVA *p*-value < 0.05 considered significant. Statistical analyses of relative bacterial abundance at the family and phylum taxonomic levels were accomplished in a stepwise fashion. First, separate multifactor analyses were performed for each experimental factor (time point, basal diet, and CP supplement) using MaAsLin2, which fits a general linear model to each microbial feature and controls for covariates as fixed effects in the model [34]. This analysis provides the overall main effects of time point, basal diet, or cocoa polyphenol supplement using the complete dataset. Subsequently, metagenomeSeq was employed to explore further differences in the relative abundance of bacteria for specific experimental conditions using datasets segregated by time point and then experimental diet in a pairwise fashion; a zero-inflated Gaussian fit was employed with a false discovery rate (FDR)-adjusted *p*-value < 0.05 considered statistically significant. The heat tree analysis leverages the hierarchical structure of taxonomic classifications to quantitatively (using median abundance) and statistically (nonparametric Wilcoxon rank sum test) depict taxonomic differences between microbial communities [35].

The functional capacity of the detected bacteria was predicted using Tax4Fun to generate gene abundance tables based on a minimum 16S rRNA sequence similarity. These tables were then processed using the Microbiome Analyst Shotgun Data Profiling module, with data filtering and normalization as described above. The dataset was then analyzed using MaAsLin2 to identify KEGG orthology terms that were differentially abundant for each of the three experimental factors: time point, basal diet, and cocoa supplement (FDR *p* < 0.05). The lists of significant terms were then subjected to pathway association analysis using the globaltest algorithm to identify enriched functional pathways (*p* < 0.05). Last, to compare the shift in microbiome composition longitudinally, both the taxonomy and functional datasets were analyzed by non-metric multi-dimensional scaling (NMDS) using the Bray–Curtis dissimilarity method and then visualized as the first or second axis with respect to time for pairwise comparisons between basal diets (AIN vs. TWD) or supplement (CON vs. CP).

### 2.7. Other Statistical Analyses

For statistical analyses not described above, a linear mixed model (LMM) was used, with cage as a nested, random factor (as appropriate), employing the restricted maximum likelihood estimation method, along with Tukey’s HSD post hoc test for multiple comparisons (JMP v.17.1.0, SAS Institute, Cary, NC, USA). Outliers were identified by the robust outlier test (ROUT) with a *Q* value of 0.1% (Prism). Data that did not meet normality or variance assumptions for the parametric test were transformed using log_10_ or square root transformation. For data that were not normally distributed or for which transformation did not resolve issues with normality or variance, a nonparametric Steel–Dwass test was used to determine the main effects of basal diet and CP supplementation (JMP). However, if the results of the nonparametric Steel–Dwass tests did not differ from the original LMM analyses for significant outcomes, the original LMM test results are reported, as the mixed model accounts for potential cage effects. Mortality incidence was analyzed using Fisher’s Exact Test to compare between groups (Prism), and differences in survival curves were assessed using the Kaplan–Meier survival analysis (Prism). Food and energy intakes were evaluated on a per-cage basis. For all analyses, an adjusted *p* < 0.05 was considered a significant effect of the test variable. Final sample sizes are reported in Table S2.

## 3. Results

### 3.1. Mortality Due to AOM/DSS Treatment and Adenoma Development

**Figure 1:**
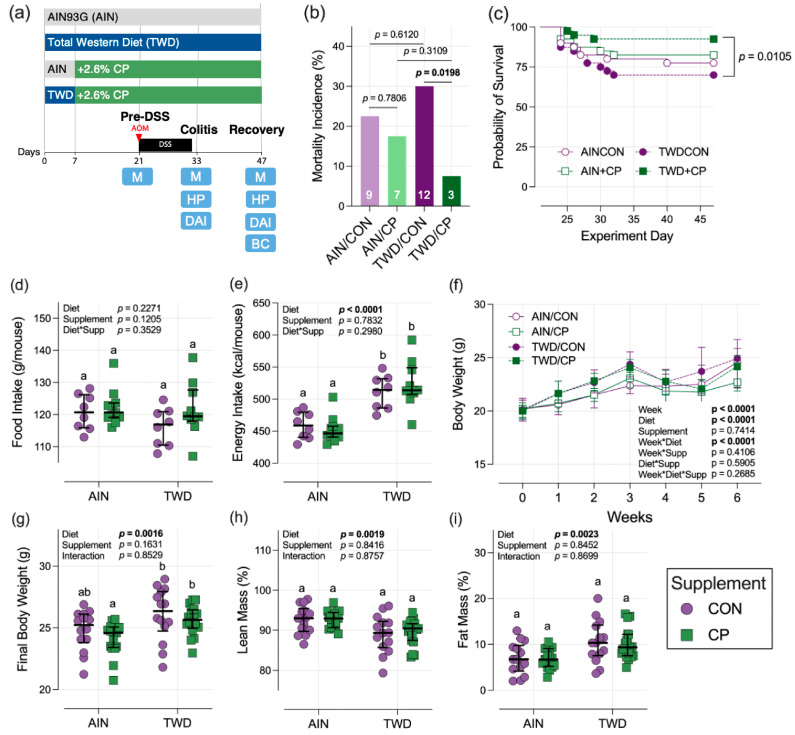
Study design, mortality, food and energy intakes, body weight, and body composition. (**a**) Diagram depicting study design. Basal diets are represented as gray (AIN) and blue (TWD) with the addition of 2.6% (*w*/*w*) CocoaVia Cardio Health Powder, depicted in green. Experimental time points are labeled pre-DSS (day 21), colitis (day 33), and recovery (day 47). Endpoints are shown below the timeline: fecal microbiome profiling and metagenomics (M), disease activity index (DAI), histopathology (HP), and body composition (BC). Other abbreviations: azoxymethane (AOM) and dextran sodium sulfate (DSS). (**b**) Mortality incidence is shown as the percentage of total mice in each experimental group after day 21 (*n* = 40). Values within the bars indicate the number of deaths. (**c**) Percent survival after AOM injection on day 21, with only significant *p*-values shown. (**d**,**e**) Estimated total food and energy intake per mouse per cage (*n* = 8 to 11). Longitudinal food and energy intake data in Figure S2. (**f**) Weekly body weight throughout the study (*n* = 28 to 37 day 1 to 21; *n* = 28 to 26 day 22 to 32; *n* = 13 to 19 day 33 to day 47). (**g**) Final body weight on day 47 (*n* = 13 to 19). (**h**,**i**) Lean mass and fat mass as a percentage of body weight (*n* = 13 to 19). For (**d**–**i**), all data are shown for individual measurements (except for (**f**), which displays the median for each group at each weekly measure) with the median ± interquartile range. The inset tables display the main effects of diet, supplement, and their interaction in the statistical model. Different letters in the experimental groups indicate that they are significantly different (*p* < 0.05), as determined as described in the Materials and Methods.

A modest level of mortality in this mouse model of CAC, generally ~15%, is expected due to the acute hepatotoxic effects of AOM and severe colitis symptoms (e.g., weight loss) requiring preemptive euthanasia. Accordingly, the initial group size, informed by an a priori power analysis, accounts for this expected loss. Interestingly, we observed higher than anticipated mortality in some experimental groups in this study (Table S2). Most notably, there was a significant difference in mortality between the TWD/CP group, with 7.5% mortality, and the TWD/CON group, with 30% mortality (*p* = 0.0198) (Figure 1b), suggesting that CP supplementation conferred protection in TWD-fed mice. Furthermore, Kaplan–Meier survival analysis indicated that the survival of mice in the TWD/CP group was significantly greater (*p* = 0.015) than that of their TWD/CON counterparts (Figure 1c). The AOM+DSS protocol employed in this study did result in the rapid development of adenomas in some mice by the recovery time point, 3.5 weeks after disease initiation (Figure S1; Table S2). However, the incidence of adenoma in colon tissues was not statistically different between the basal diet and the CP supplement.

### 3.2. Food and Energy Intake, Body Weight, and Body Composition

Although there was not a difference in total estimated food intake, mice consuming TWD had a significantly higher total energy intake compared to mice consuming AIN (*p* < 0.0001) due to the higher energy density of the TWD (Figure 1d,e and Figure S2). CP supplementation did not affect the estimated food or energy intakes for mice provided either basal diet. Basal diet significantly affected final body weight, with mice consuming TWD weighing on average, 5.16% more than mice consuming AIN (*p* = 0.0016) (Figure 1g). Body composition also differed between the basal diets, with mice consuming TWD having, on average, 3.3 g more fat mass than their AIN counterparts (*p* = 0.0023) (Figure 1h,i). Alternatively, CP consumption had no apparent effect on final body weight or body composition.

### 3.3. Organ Weights

Relative spleen weights between groups were similar (Figure S3a). Liver weights relative to body weight were 6.7% greater in mice consuming the TWD (diet main effect *p* = 0.0289) (Figure S3b). An interaction effect for liver weights was also observed (*p* = 0.0207), with AIN/CP exhibiting increased weight compared to AIN/CON and TWD/CP showing decreased weight compared to TWD/CON. CP supplementation significantly decreased relative kidney mass by 3.1% (*p* = 0.0293) and increased the relative mass of the cecum contents by 20.7% (*p* = 0.0103), with no effect of basal diet on either of these endpoints (Figure S3c,d).

### 3.4. Disease Activity Index, Histopathology, and Colon Length

**Figure 2:**
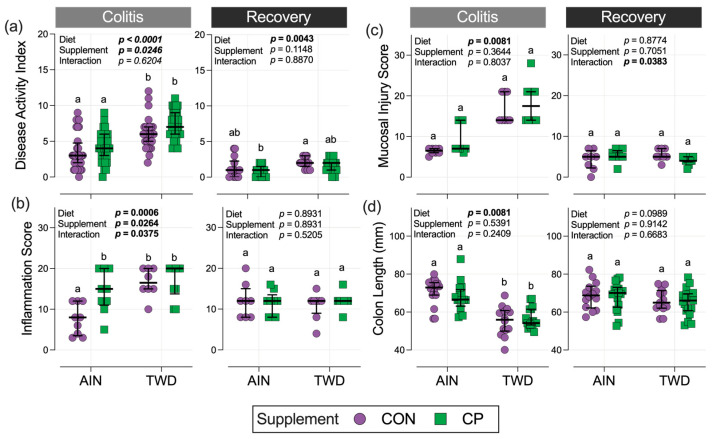
Disease activity index, colon histopathology, and colon lengths. (**a**) Disease activity index (DAI) scores (*n* = 28 to 36 for colitis, *n* = 13 to 19 for recovery); (**b**) histopathology inflammation scores and (**c**) histopathology mucosal injury scores (*n* = 8 to 10 for colitis, *n* = 8 to 11 for recovery); (**d**) colon lengths (*n* = 14 to 18 for colitis, *n* = 14 to 19 for recovery) during active colitis on day 33 and after recovery on day 47. Data are shown as individual values with the median ± interquartile range. The inset tables display the main effects of diet, supplement, and their interaction in the statistical model. Different letters for experimental groups indicate they are significantly different (*p* < 0.05), determined as described in the Materials and Methods.

Consistent with our previous work [5,16,17], during active colitis, the DAI score for mice fed the TWD was increased by an average of 2.9 points compared to their counterparts fed the AIN diet, regardless of CP supplementation (diet main effect, *p* < 0.0001) (Figure 2a). This inflammation-promoting effect of the TWD persisted through the recovery time point, where TWD-fed mice scored 0.81 points higher, on average, than AIN-fed mice (diet main effect, *p* = 0.0043). Surprisingly, a significant main effect of CP supplementation was observed during active colitis (CP main effect *p* = 0.0246) as CP increased the DAI by 1.2 points on average, irrespective of the basal diet. However, this effect was not evident at the recovery time point. Also, no significant pairwise differences between CON and CP groups were noted within each basal diet group (Figure 2a).

Histopathological analysis of colon tissues to determine the extent of inflammation in the mucosa indicated a similar trend as the DAI index, with mucosal inflammation scores 53.8% higher for mice fed the TWD compared to AIN at the colitis time point (diet main effect *p* = 0.0006) (Figure 2b). Notably, dietary supplementation with CP in AIN-fed mice caused a marked 89.7% increase in mucosal inflammation score during active colitis compared to their AIN/CON counterparts (*p* = 0.0149), whereas CP did not affect inflammation scores in TWD-fed mice. At recovery, inflammation scores were not affected by the basal diet or CP supplementation. Mucosal injury scores at colitis were likewise increased 1.3-fold in mice consuming the TWD (diet main effect *p* = 0.0081) but differed from the inflammation scores in that they were unaffected by CP consumption (Figure 2c). Mucosal injury at the colitis time point was not affected by diet or supplement; however, a small interaction effect was observed (*p* = 0.0383).

Interestingly, colon length at the colitis time point reflected the effects of basal diet, with colons from the AIN group being 23% longer than those of mice in the TWD group (diet main effect *p* = 0.0081) (Figure 2d). There was no effect of CP intervention on colon length despite increased DAI and histopathology scores of inflammation. At recovery, colon lengths were comparable between the experimental groups, with no significant differences due to diet or supplementation.

### 3.5. Gene Expression Analysis

**Figure 3:**
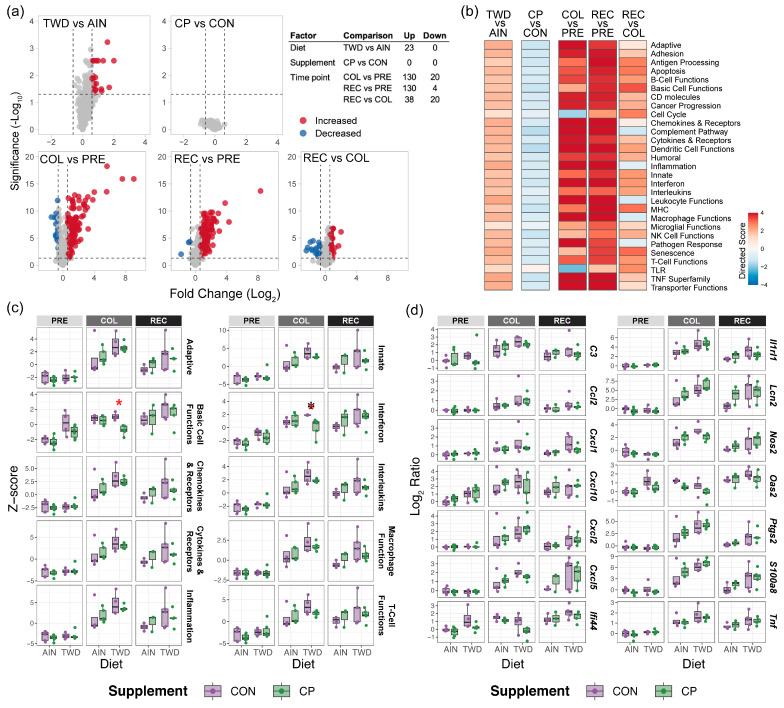
NanoString gene expression analysis for time point, diet, and supplement in mouse colon mucosa. (**a**) Volcano plots depicting significant differentially expressed genes (DEGs) for each experimental factor with fold change > 1.5 and BH-adjusted *q*-value < 0.05. (**b**) Directed gene set significance scores for immune-related pathways included in the NanoString PanCancer Immune Profiling gene panel. (**c**) Pathway Z scores are shown as Tukey box plots (*n* = 3 to 6) for selected immune and cancer pathways. Figure S5 shows a heatmap for all individual samples for all pathways included in the gene panel. *, significantly different for CP vs. CON within the basal diet group as determined by general linear model regression with Tukey’s HSD post hoc test with FDR-adjusted *p*-value < 0.05. Full statistical results are available in File S1. (**d**) Log_2_ ratios for select genes of interest with respect to AIN/CON at the pre-DSS time point as the reference. Results of nSolver statistical analyses are provided in File S1.

To evaluate how CP supplementation influenced inflammation-related gene expression in the colon, we analyzed transcriptional profiles of 750 immune- and cancer-related genes, comparing responses across time points (pre-DSS, colitis, recovery) and basal diet (AIN or TWD). The greatest number of differentially expressed genes (DEGs) was observed during colitis (COL vs. PRE), with 130 upregulated and 20 downregulated genes, highlighting a strong transcriptional response during active disease (Figure 3a and Figure S4; File S1). This response persisted at the recovery time point (REC vs. PRE), with 130 genes upregulated and only 4 downregulated. A direct comparison of REC vs. COL revealed 38 upregulated and 20 downregulated genes, indicating that although recovery was associated with a resolution of some gene expression changes, many alterations persisted. With respect to diet, TWD-fed mice showed a modest but distinct transcriptional signature compared to AIN-fed mice at the pre-DSS time point, with 23 upregulated DEGs and 0 downregulated genes. In contrast, there were no DEGs identified when comparing CP vs. CON-fed mice, indicating a minimal effect of the dietary supplement on cancer and immune gene expression at the selected significance thresholds.

Gene set analysis (GSA) revealed that the strongest transcriptional response occurred during colitis and recovery, characterized by the widespread activation of pathways related to inflammation, innate and adaptive immunity, interferon signaling, and cytokine production (Figure 3b). These effects were notably more pronounced in mice fed the TWD compared to their AIN counterparts, indicated by positive GSA scores for this comparison, irrespective of time point or supplement. However, supplementation with CP did not result in meaningful modulation of pathway activity, consistent with the DEG findings.

Similar trends were evident for the pathway Z scores, which provide an orthogonal assessment of pathway-level gene expression based on the first principal component of normalized expression (Figure 3c and Figure S5). A similar pattern emerged, with marked activation of immune-related pathways during colitis and recovery, particularly in mice fed the TWD. Several pathways, including those involved in interferon signaling, antigen processing, and T-cell function, were consistently elevated in TWD-fed mice relative to AIN. While no individual DEGs were identified when comparing CP-fed mice to their CON counterparts, the Z scores were significantly reduced in mice fed the basal TWD with CP vs. TWD/CON for the interferon pathway (*p* = 0.0400) and basic cell function (*p* = 0.0014) (Figure 3c).

Lastly, Figure 3d presents the expression patterns of selected genes with strong biological relevance and statistical significance (BH-adjusted *q* < 0.05, with a fold change > 1.5) (File S1). Genes such as *C3*, *Ccl2*, *Cxcl1*, *Ifi44*, *Lcn2*, *S100a8*, and *Tnf* were strongly induced during colitis, mirroring previously reported inflammation-associated transcriptional responses. Many of these genes remained elevated during recovery, although to a lesser extent. Among diet-responsive genes, *Cxcl10* and *Oas2* were elevated in TWD-fed mice compared to AIN-fed mice at baseline. No significant changes in expression were observed for CP vs. CON for these biomarker genes across all time points.

### 3.6. Relative Abundance of Bacteria in Fecal Microbiomes

#### 3.6.1. Sequencing Results, Filtering, and Rarefaction

Sequencing resulted in 10.1 × 10^6^ reads of the 16S rRNA V4 region amplicon for all samples combined. Following quality filtering and chimera removal, 6.6 × 10^6^ sequences were assigned to ASVs (Silva database version 138 SSU) using QIIME2, resulting in an average of 28,632 sequences per sample assigned to 663 ASVs. The resulting sequence count data aligned to taxonomy are provided in File S2. After filtering for low prevalence and variance as described in our methods, the sequence library was rarefied to a sequencing depth of ~6000 sequences (Figure S6). Given the multi-level experimental model considering basal diet (AIN and TWD), cocoa polyphenol supplement (CON and CP), and time point (pre-DSS, colitis, and recovery), microbiome analyses were performed in a stepwise fashion, first considering the main effects of these experimental factors (Figures S8 and S9) followed by pairwise comparisons, as appropriate, of the diet and supplement groups within each time point.

#### 3.6.2. Microbiome Composition Changes Across Disease Progression and Recovery

**Figure 4:**
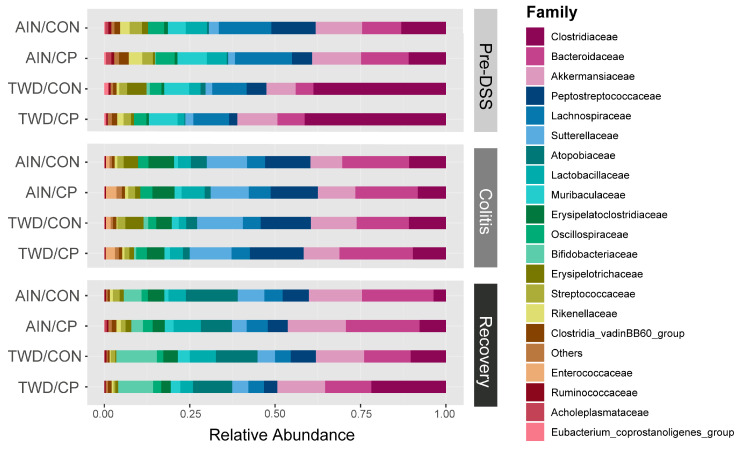
Taxonomic classification of mouse fecal microbiome. Data are shown as average relative normalized abundance of bacteria at the family level for each diet/supplement group within each time point.

Substantial shifts in the microbiome were observed in all experimental groups as mice transitioned from healthy conditions to chemically induced colitis and various states of recovery (Figure 4 and Figure S7). At the phylum level, Firmicutes decreased from 65% pre-DSS to 52.4% during colitis (*p* = 7.62 × 10^−13^) and 39.2% at recovery (*p* = 3.04 × 10^−34^), while Proteobacteria (primarily Sutterellaceae) rose from 2.3% to 12% during colitis (*p* = 2.8 × 10^−49^), declining to 5.3% at recovery (*p* = 1.85 × 10^−11^). Actinobacteria, mainly Atopobiaceae and Bifidobacteriaceae, remained low during colitis but rose to 19.3% at recovery (*p* = 2.38 × 10^−28^) (File S3).

At the family level (Figure S8a), Clostridiaceae dropped from 26% pre-DSS to 9.7% during colitis (*p* = 6.34 × 10^−4^), while Bacteroidaceae increased from 9.6% to 18.6% (*p* = 2.67 × 10^−5^). Colitis also led to increases in Peptostreptococcaceae (6.7% to 14.2%, *p* = 9.84 × 10^−15^), Erysipelotrichaceae (2.3% to 2.9%, *p* = 5.37 × 10^−9^), and Enterococcaceae (0.02% to 2.1%, *p* = 3.73 × 10^−56^). Declines were noted in Lachnospiraceae (13.2% to 5.6%, *p* = 8.18 × 10^−34^), Akkermansiaceae (12.1% to 10.8%, *p* = 1.98 × 10^−4^), Muribaculaceae (7.4% to 1.6%, *p* = 2.86 × 10^−24^), and Streptococcaceae (2.8% to 1.9%, *p* = 1.14 × 10^−8^). At recovery, several taxa, including Akkermansiaceae, Enterococcaceae, and Peptostreptococcaceae, returned to their baseline levels. Erysipelotrichaceae fell below baseline (1.1%, *p* = 3.89 × 10^−9^). Clostridiaceae and Bacteroidaceae partially rebounded (11.2%, *p* = 3.23 × 10^−4^ and 17.5%, *p* = 1.75 × 10^−5^, respectively), while Lachnospiraceae declined further to 5.3% (*p* = 3.71 × 10^−33^).

#### 3.6.3. Impact of Basal Diet on the Microbiome

**Figure 5:**
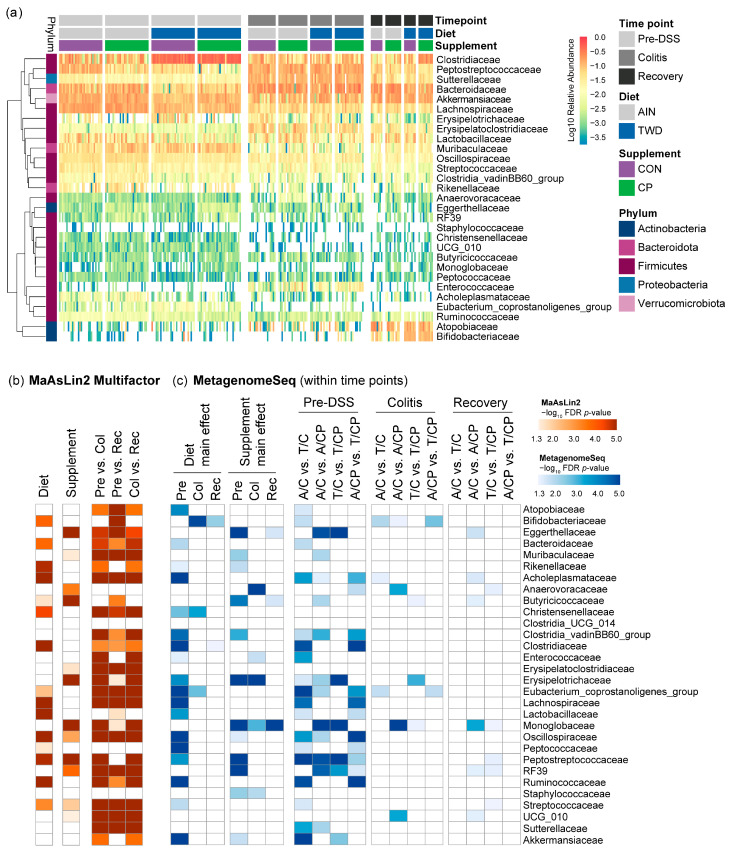
Relative abundance of fecal microbiome at the family taxonomic level with summary of results of MaAsLin2 Multifactor and metagenomeSeq analyses. (**a**) Unsupervised hierarchical cluster analysis was used to visualize the log_10_ relative abundance of taxa, clustering by Euclidean distance with average linkage (*n* = 30 for pre-DSS, *n* = 15 to 20 for colitis, and *n* = 8 to 11 for recovery). (**b**) The summary plot shows the log_10_ FDR-adjusted *p*-value obtained from MaAsLin2 analysis of the fecal microbiome profile for the main effects of diet and supplement over the entire experiment, as well as time point pairwise comparisons. (**c**) The summary plot shows the log_10_ FDR-adjusted *p*-values obtained from metagenomeSeq analyses of fecal microbiome profiles for diet and supplement main effects within each time point, as well as pairwise comparisons within each time point. An effect was considered significant with an FDR-adjusted *p*-value < 0.05, shown in an orange color scale for MaAsLin2 results (**b**) and blue color scale for metagenomeSeq results. (**c**) Complete results of MaAsLin2 and metagenomeSeq statistical analyses are provided in File S3.

**Figure 6:**
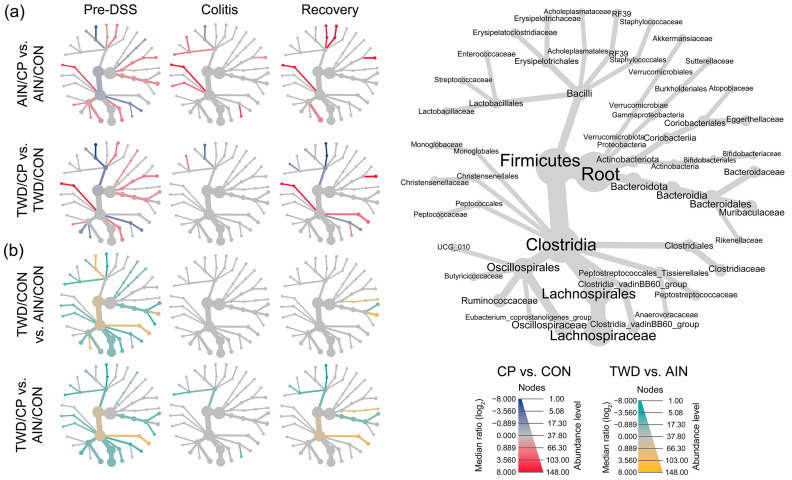
Fecal microbiome community structures are shown as heat trees, displaying the relative abundance ratios for select pairwise comparisons to show the effects of basal diet and CP supplementation at each experimental time point. (**a**) Comparisons of CP-supplemented diets with CON diets for both AIN and TWD basal diets (blue-to-red color bars, with red indicating greater relative abundance in CP-supplemented groups). (**b**) Comparisons of basal diet, with or without CP supplementation (green-to-yellow color bars, with yellow indicating greater abundance in TWD-fed groups).

**Figure 7:**
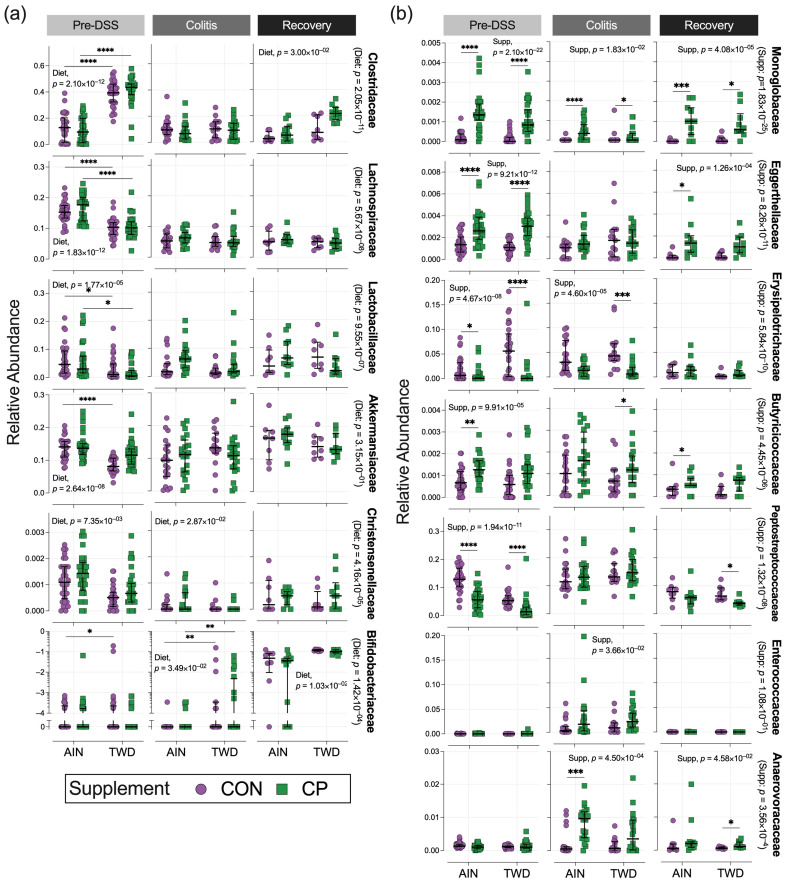
Relative abundance of select bacteria families primarily affected by basal diet or CP. (**a**) Bacterial families affected by basal diet: Clostridiaceae, Lachnospiraceae, Lactobacillaceae, Christensenellaceae, and Bifidobacteriaceae. (**b**) Bacterial families affected by CP consumption: Monoglobaceae, Eggerthellaceae, Erysipelotrichaceae, Butyricicoccaceae, Peptostreptococcaceae, Enterococcaceae, and Anaerovoracaceae. Data points show relative abundance values for individual cages, plotted with the median ± interquartile range (*n* = 30 for pre-DSS, *n* = 15 to 20 for colitis, and *n* = 8 to 11 for recovery). Bifidobacteriaceae data are shown as log_10_ ratios for better visualization. Plots show main effects within the time point or significant pairwise comparisons by diet, indicated as follows: *, *p* < 0.05; **, *p* < 0.01; ***, *p* < 0.001; ****, *p* < 0.0001. *p*-values for diet main effects overall are shown with each bacteria family label. Statistical methods are outlined in the Materials and Methods Section. Complete results of MaAsLin2 and metagenomeSeq statistical analyses are provided in File S3.

Prior to DSS treatment, the TWD significantly altered microbial composition (Figure 4, Figure 5, Figure 6, Figure 7 and Figure S9a). It increased Clostridiaceae (12% to 40%, *p* = 2.1 × 10^−12^) and decreased Lachnospiraceae (16% to 10.3%, *p* = 1.83 × 10^−12^), Lactobacillaceae (6% to 2.7%, *p* = 1.77 × 10^−6^), and Akkermansiaceae (14% to 10.2%, *p* = 2.64 × 10^−8^). The rare family Christensenellaceae was also reduced (0.13% to 0.07%, *p* = 7.35 × 10^−3^). At the colitis stage, only Christensenellaceae maintained a diet-related difference (0.02% AIN vs. 0.01% TWD, *p* = 0.0287). At recovery, Clostridiaceae increased again in TWD-fed mice (*p* = 0.0300), and Bifidobacteriaceae showed a significant rise in TWD groups at both colitis (0.004% to 0.99%, *p* = 0.0349) and recovery (4.18% to 10.98%, *p* = 0.0103), a novel observation in our studies.

#### 3.6.4. Effects of CP Supplementation

Two weeks of CP supplementation induced notable shifts (Figure 4, Figure 5, Figure 6 and Figure 7, Figure S9b), particularly in the rare bacteria family Monoglobaceae (*Monoglobus* spp.), which increased significantly at all stages: pre-DSS (0.15% AIN/CP vs. 0.02% AIN/CON, *p* = 1.71 × 10^−6^), colitis (0.03% vs. 0.01%, *p* = 0.0183), and recovery (0.10% vs. 0.01%, *p* = 4.08 × 10^−5^) (Figure 7b). These effects were more pronounced in mice fed the AIN vs. TWD basal diet. Despite low abundance overall, Monoglobaceae consistently responded to CP. Eggerthellaceae was also increased by CP at pre-DSS (0.30% vs. 0.13%, *p* = 9.21 × 10^−12^) and during recovery (0.14% vs. 0.02%, *p* = 1.26 × 10^−4^), but not during colitis. Other taxa affected pre-DSS included Erysipelotrichaceae (3.7% to 0.8%, *p* = 4.67 × 10^−8^), Butyricicoccaceae (0.07% to 0.13%, *p* = 9.91 × 10^−5^), and Peptostreptococcaceae (9.4% to 4.1%, *p* = 1.94 × 10^−11^). During colitis, Erysipelotrichaceae remained lower in TWD/CP mice (*p* = 7.19 × 10^−4^), and Enterococcaceae (*p* = 0.0366) and Anaerovoracaceae (*Eubacterium nodatum group*, *p* = 4.5 × 10^−4^) increased. By recovery, Anaerovoracaceae remained elevated in CP-fed groups (0.28% vs. 0.12%, *p* = 0.0456), while Enterococcaceae normalized.

#### 3.6.5. Firmicutes/Bacteroidota (F:B) Ratio Dynamics

As the disease progressed, the F:B ratio decreased, reaching its lowest point at recovery, primarily driven by a decline in Firmicutes (time point effect, *p* < 0.0001; Figure S8b). Prior to DSS, TWD significantly increased the F:B ratio (*p* < 0.0001), an effect that disappeared during colitis and returned at recovery (*p* = 0.0026). CP supplementation reduced the F:B ratio prior to DSS treatment (*p* < 0.0001) but had no significant impact during colitis or recovery.

### 3.7. Alpha and Beta Diversity of Fecal Microbiomes

**Figure 8:**
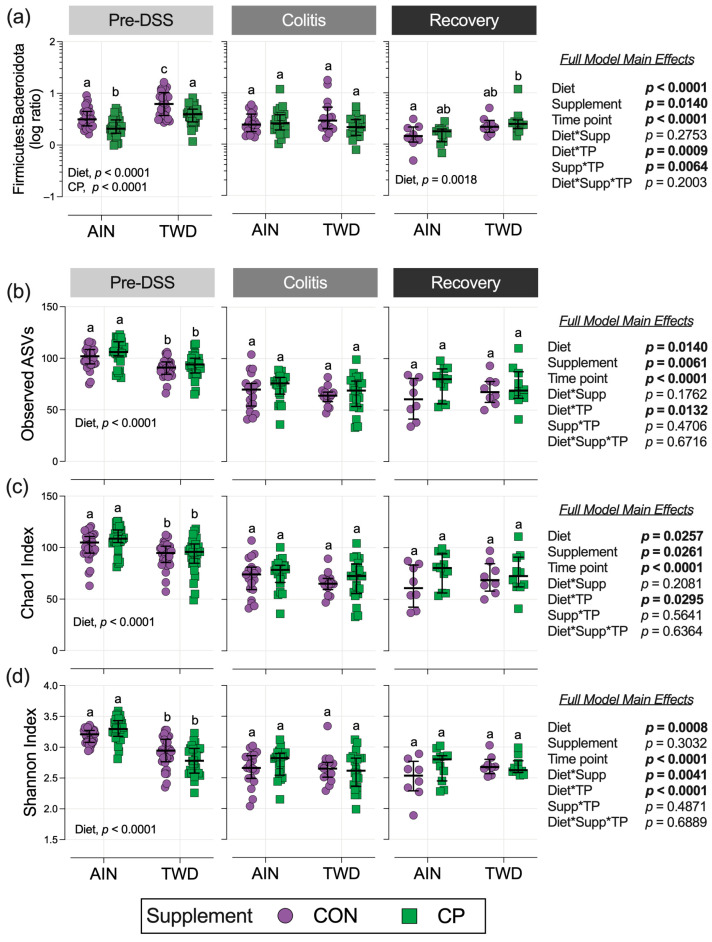
Firmicutes-to-Bacteroidetes ratio and alpha diversity. (**a**) Firmicutes-to-Bacteroidetes ratio for each experimental time point. Ratios were calculated using normalized count data for each phylum and are shown on a log_10_ scale. (**b**–**d**) Alpha diversity of mouse fecal microbiome at each experimental time point, shown as the observed ASVs (**b**), Chao 1 index (**c**), and Shannon index (**d**). Data points represent individual cages, plotted with the median ± interquartile range (*n* = 30 for pre-DSS, *n* = 15 to 20 for colitis, and *n* = 8 to 11 for recovery). Different letters above groups within each time point indicate they are significantly different (*p* < 0.05), calculated according to the methods in the Materials and Methods. The inset tables display the statistical model’s main effects and interactions for each alpha diversity measure, as well as the significant main effects of either basal diet or CP supplementation within each time point. For results of alpha diversity pairwise comparisons by experimental group within time points, see Table S3.

Alpha diversity decreased throughout this study, with both colitis and recovery time points having a significantly lower α-diversity than the pre-DSS time point when measured by the observed ASVs, Chao1 index, and Shannon index (time point main effect *p* < 0.0001 for all measures; Figure 8b–d). Alpha diversity was significantly reduced in TWD-fed mice compared to AIN-fed mice by 13 to 15% for all measures at the pre-DSS time point (diet main effect *p* < 0.0001 for each measure), irrespective of dietary CP. However, this effect of TWD was not apparent at the time points of colitis or recovery. CP supplementation consistently increased α-diversity compared to CON groups when measured by the observed ASVs and Chao 1 index over the entire study period (main effect of supplement *p* = 0.0061 and *p* = 0.0261, respectively) (Figure 8b,c). However, no significant pairwise effects of CP supplementation versus CON were noted within either basal diet group at each time point (Table S3).

**Figure 9:**
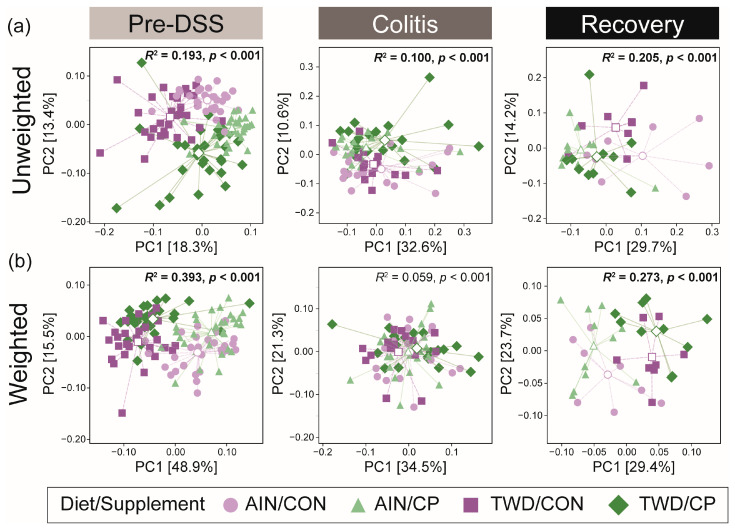
UniFrac beta diversity of mouse fecal microbiome at each experimental time point. Beta diversity is shown as (**a**) unweighted and (**b**) weighted UniFrac distances, reported as principal coordinate analysis plots using the first two coordinates (*n* = 30 for pre-DSS; *n* = 15 to 20 for colitis; *n* = 8 to 11 for recovery). Variations associated with PC1 and PC2 are shown in the axis labels, and segments connect each sample to the group centroid, marked by a white symbol. The overall PERMANOVA R2 and *p*-value are shown, with pairwise results provided in Table S4.

Beta diversity was assessed using both weighted and unweighted UniFrac distances, which account for the relative abundance of all taxa and the presence of rare taxa, respectively. Beta diversity analysis showed that, prior to the induction of colitis, microbiome composition differed significantly across experimental treatment groups, as measured by both unweighted and weighted UniFrac metrics (Figure 9). These differences were evident for supplement type, with significant pairwise PERMANOVA results for unweighted UniFrac for AIN/CON vs. AIN/CP (*R*^2^ = 0.126, *p* < 0.001) and TWD/CON vs. TWD/CP (*R*^2^ = 0.119, *p* < 0.001) (Table S4). Additionally, a distinction was apparent between the basal diets for the comparison of AIN/CON and TWD/CON (*R*^2^ = 0.113, *p* < 0.001). Similarly distinct microbiome patterns were evident for weighted UniFrac distances, accounting for the relative abundance of taxa in the microbiome, with significant measures for AIN/CON vs. AIN/CP (*R*^2^ = 0.116, *p* < 0.001) and TWD/CON vs. TWD/CP (*R*^2^ = 0.337, *p* < 0.001). Again, microbiomes were also distinct for the basal diets, with AIN/CON vs. TWD/CON (*R*^2^ = 0.352, *p* < 0.001) and AIN/CP vs. TWD/CON (*R*^2^ = 0.337, *p* < 0.001).

Alternatively, during colitis, the fecal microbiomes of mice fed either basal diet or either supplement were not notably distinct when measured by weighted or unweighted UniFrac beta diversity (Figure 9, Table S4), with all *R*^2^ ≤ 0.10 and/or *p* > 0.05 (Figure 9; Table S4). By the recovery time point, however, distinct microbiomes were again apparent, though to a lesser extent than prior to the induction of colitis. CP-supplemented samples clustered separately from their basal diet control groups when measured by unweighted UniFrac (*R*^2^ = 0.194, *p* = 0.012 for AIN/CON vs. AIN/CP and *R*^2^ = 0.138, *p* = 0.012 for TWD/CON vs. TWD/CP), again indicating that the distinctiveness was likely driven by rare taxa. On the other hand, basal diet had a greater effect on the weighted distances, with *R*^2^ = 0.0.166, *p* = 0.009 for AIN/CON vs. TWD/CON and *R*^2^ = 0.0.279, *p* = 0.002 for AIN/CP vs. TWD/CP (Table S4).

### 3.8. Predicted Functional Metagenomics and Longitudinal Analyses

Prediction of functional potential revealed significant differences in KEGG orthology terms across time points, as well as in response to the basal diet and CP supplementation. At the pre-DSS stage, the TWD was associated with marked depletion in KEGG level 2 terms related to glycan biosynthesis and metabolism, secondary metabolite biosynthesis, and amino acid metabolism compared to the AIN diet (Figure S10). These differences were largely independent of CP supplementation. During colitis, few KEGG level 2 terms differed by diet. By recovery, some terms returned to pre-DSS levels, particularly those related to nucleotide metabolism, glycan metabolism, and energy metabolism.

CP supplementation affected a subset of level 2 KEGG terms prior to DSS administration, with the most pronounced changes observed in the TWD/CP versus TWD/CON comparison. In TWD/CP mice, nucleotide metabolism and terpenoid/polyketide metabolism terms were reduced, while glycan and energy metabolism terms were more abundant. During colitis, CP led to modest enrichment of carbohydrate and amino acid metabolism terms in the TWD/CP group. No significant differences were detected at recovery.

Significant level 2 KEGG terms (identified via MaAsLin2) were mapped to level 3 pathways to assess broader functional changes (Figure S11). The pre-DSS microbiome exhibited the most distinct profile, characterized by unique enrichments in pantothenate and CoA biosynthesis, carbon fixation in photosynthetic organisms, and fructose and mannose metabolism, compared to colitis, and carbon fixation in prokaryotes, compared to recovery (Figure S11a,c). During colitis, the alanine, aspartate, and glutamate metabolism pathway became the most enriched, replacing amino sugar and nucleotide sugar metabolism, which had previously dominated before DSS exposure (Figure S11b).

Analysis of KEGG level 3 pathways revealed that many were significantly enriched in the TWD compared to the AIN diet (Figure S11d). Notably, pyruvate metabolism and galactose metabolism showed strong enrichment, along with vitamin B6 metabolism, which was among the most enriched despite being associated with relatively few level 2 terms. Other pathways of interest included propanoate metabolism and the metabolism of starch and sucrose.

CP supplementation had fewer effects on level 3 pathways (Figure S11e). Lysine biosynthesis was the most significantly enriched in the CP-fed groups compared to controls. Additional enriched pathways included lipopolysaccharide biosynthesis, vitamin B6 metabolism, and oxidative phosphorylation. Monobactam biosynthesis emerged as a uniquely enriched pathway associated specifically with CP supplementation.

**Figure 10:**
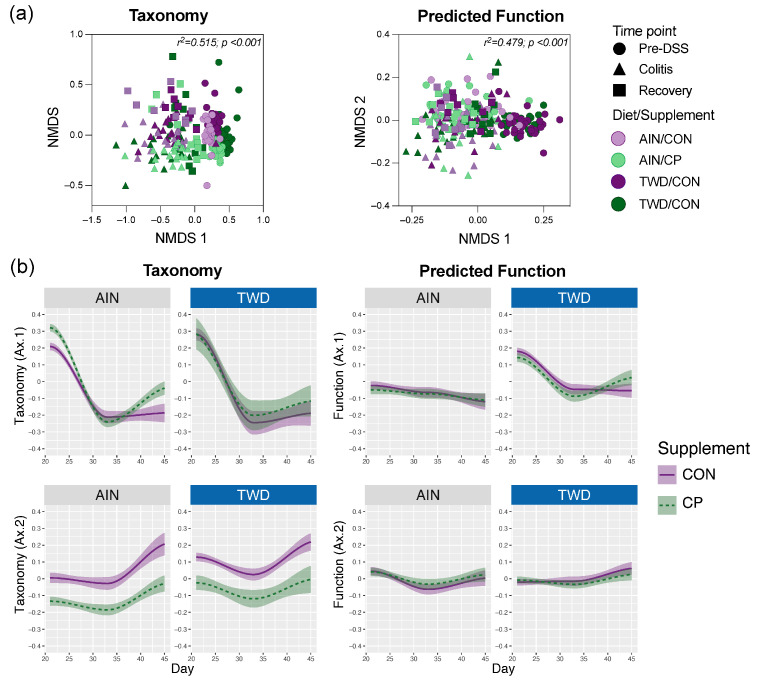
Longitudinal analysis of mouse fecal microbiome taxonomy and functional capacity. (**a**) Taxonomic and functional beta diversity of fecal microbiomes for all experimental groups for all time points shown as NMDS ordination plots of the Bray–Curtis dissimilarity measure of either ASV abundance for taxonomy or KEGG term abundance for functional capacity (*n* = 30 for pre-DSS; *n* = 15 to 20 for colitis; *n* = 8 to 11 for recovery). (**b**) Longitudinal variation is shown as the first and second dimensions plotted over the experimental days, starting on day 20, for taxonomic and functional diversity. Loess-smoothed trajectories of microbiomes from each experimental day are plotted, with purple indicating CON diets and green indicating CP-supplemented diets. Shaded areas represent the 95% confidence interval.

Longitudinal Bray–Curtis β-diversity analysis was conducted on both taxonomic composition and predicted function, with samples grouped by time point (Figure 10). NMDS plots (Figure 10a) showed clear separation of pre-DSS samples from those collected during colitis and recovery. At the pre-DSS stage, taxonomic profiles were separated by both diet (axis 1) and supplement (axis 2), while predicted functional profiles were separated only by diet. No distinct clustering by diet or supplement was observed during colitis; however, recovery samples began to re-cluster by diet. Longitudinal trajectories along NMDS axis 1 (Figure 10b) illustrated temporal dynamics. CP supplementation led to distinct shifts in taxonomic composition over time in both dietary backgrounds. In contrast, predicted functional profiles remained relatively stable, with only modest variation during the colitis and recovery periods.

## 4. Discussion

Cocoa polyphenols have been extensively studied for their anti-inflammatory and gut microbiome-modulating effects, both in vitro and in vivo as (reviewed in [36,37]). In this study, we tested the hypothesis that CP supplementation would reduce inflammation and beneficially alter the gut microbiome in a mouse model of colitis induced by AOM and DSS. Contrary to expectations, 2.6% (*w*/*w*) CocoaVia™ Cardio Health Powder did not mitigate inflammation, as assessed by DAI scores or histopathology. We observed increased DAI scores and inflammation in both basal diets with CP supplementation. These findings challenge previous studies reporting reduced inflammation and tumorigenesis with cocoa intake [20,21,22,38,39,40]. Nonetheless, CP intake significantly altered gut microbiome composition, with distinct profiles observed through disease induction and recovery. This suggests that cocoa polyphenols exert microbiome-modulating effects independent of their ability to suppress colitis symptoms. These results highlight the complexity of dietary interventions in modulating inflammation and underscore the need to consider dietary context, such as a Western-style diet, which itself promotes inflammation and microbial dysbiosis.

While the pro-inflammatory effects of the TWD were consistent with previous work [5,16,41,42], the increase in inflammation observed with CP supplementation was unexpected. Cocoa polyphenols have been shown to suppress inflammatory pathways, including NF-κB activation and cytokine gene expression, in vitro and in some in vivo models [20,21,22,38,39,40]. Discrepancies between our findings and those studies likely reflect differences in experimental design. For instance, two studies by Saadatdoust et al. and Pandurangan et al., which used BALB/c female mice in a three-cycle DSS model with AIN-based diets supplemented with 5% or 10% cocoa, reported decreased tumorigenesis and inflammation [21,22]. In contrast, our model used male C57BL/6J mice, included a 21-day pre-feeding period, and employed a single 10-day cycle of 1% DSS. The basal diets also differed substantially in macronutrient composition and fiber content, which likely influenced outcomes. Furthermore, the cocoa-supplemented diets used in these studies [21,22] had significantly lower fat and protein contents than the control diets, raising the possibility that the observed effects were due to changes in macronutrients rather than the addition of cocoa. Our CP-supplemented diets maintained macronutrient parity with the controls, adjusting the corn starch and fiber to match the energy content. These methodological contrasts highlight the importance of standardizing diet composition in dietary intervention studies.

Additional studies using AOM-only models in rats have reported CP-induced reductions in aberrant crypt foci and suppression of inflammatory mediators, such as iNOS and NF-κB [40,43,44]. However, other work using a DSS-only model of colitis found that, while 5% (*w*/*w*) cocoa-enriched diet decreased oxidative stress and TNF-α levels, it did not significantly alter clinical or histological signs of colitis in rats [45]. These findings align with our results, suggesting that CPs may exert subtle biochemical or microbiome-related effects that do not translate to overt symptom reduction in acute colitis models.

One confounding factor in our study was the higher-than-expected mortality rate, particularly in control diet groups. Notably, CP supplementation improved survival in the TWD basal diet group, especially from days 24 to 32, suggesting a possible protective effect against AOM/DSS-induced mortality. This observation indicates that CP could ameliorate severe colitis caused by DSS, which may lead to early mortality. Alternatively, CP may also help to prevent liver damage induced by AOM. While this observation could reflect a benefit of CP intake, the reduced mortality also skewed the disease severity data, as severely affected control mice died before they could be assessed. Consequently, high inflammation scores in surviving CP-fed mice compared to CON may partly reflect survivor bias, emphasizing the need for cautious interpretation.

Gene expression profiling of colon mucosa samples revealed a robust transcriptional response during active colitis, consistent with our previous findings [5]. Compared to the pre-disease state, we observed differential expression of over 150 genes, including key markers of inflammation and immune activation such as *C3*, *Ccl2*, *Cxcl1*, *Ifi44*, *Lcn2*, *S100a8*, and *Tnf*. These changes corresponded to elevated activity in pathways involved in innate and adaptive immune responses, cytokine and chemokine signaling, and T-cell and macrophage function. During the recovery phase, gene expression profiles remained distinct from baseline, albeit with fewer differentially expressed genes, indicating a partial resolution of the inflammatory response. Notably, mice fed the TWD exhibited a more pro-inflammatory baseline gene expression profile prior to DSS exposure, including increased expression of *Cxcl10* and *Oas2*, compared to AIN-fed controls. CP supplementation did not significantly alter gene expression patterns at any time point relative to control diets. Although some gene set enrichment analyses suggested modest reductions in immune- and cancer-associated pathways, these were subtle (e.g., small changes in directed global significance scores) and not reflected in the number of differentially expressed genes (DEGs) after correction for multiple comparisons. This apparent discrepancy underscores the importance of pathway-level analyses, which can identify coordinated yet modest shifts in gene expression across multiple genes that may not reach statistical significance individually. Pathway Z-score analysis similarly indicated reduced activity in basic cellular processes and interferon-related pathways in CP-fed groups. Collectively, these findings align with our histopathological and clinical observations, indicating that CP supplementation has a limited impact on the mucosal immune response in this model.

Consistent with previous reports [16,46,47], alpha diversity declined during colitis and remained suppressed at the time of recovery. Notably, CP supplementation modestly increased alpha diversity prior to colitis, suggesting early microbiome enrichment, though this effect was attenuated during inflammation. Beta diversity analyses revealed substantial differences in microbial composition by diet and CP supplementation prior to disease induction, primarily driven by rare taxa. These distinct signatures re-emerged during recovery, underscoring the dynamic and context-dependent influence of CPs on the microbiome. These shifts were largely driven by rare taxa, including Monoglobaceae and Eggerthellaceae. *Monoglobus pectinilyticus*, the only described species in Monoglobaceae, ferments pectin and has been linked to flavonoid intake in other models [48,49,50,51,52], suggesting a general responsiveness to plant-derived compounds. Although pectin is low in CocoaVia™, this response may reflect broader polyphenol sensitivity. Notably, Monoglobaceae abundance was reduced in IBS patients [53], indicating a potential health-promoting role.

Eggerthellaceae, another rare bacterial family that increased with CP intake, includes members known to metabolize polyphenols, such as *Eggerthella lenta* [54]. Their relative abundance has also been shown to increase following plant-based interventions [52], supporting their putative role in metabolizing phytochemicals. Additionally, bacteria in this family may benefit colon health; as a member of the Eggerthellaceae family, *Enterorhabdus*, has been shown to be negatively correlated with IBD risk [55]. Conversely, CPs reduced the abundance of Erysipelotrichaceae and Peptostreptococcaceae, both of which have been associated with IBD and colorectal cancer [56,57,58,59,60,61,62,63]. While Erysipelotrichaceae findings are mixed, Peptostreptococcaceae has been identified as a potential opportunistic pathogen. CP-induced suppression of these families may thus have beneficial implications.

The basal diet had a strong influence on microbiome composition. TWD reduced α-diversity, shifted β-diversity, and altered the relative abundance of key taxa, consistent with prior work [16,17,64]. Notably, TWD increased Clostridiaceae, particularly *Clostridium sensu stricto 1*, which includes pathogens such as *C. perfringens* and *C. tetani*, and reduced health-associated bacterial families, including Lachnospiraceae, Akkermansiaceae, and Christensenellaceae [65,66,67,68,69]. These taxa are linked to SCFA production, mucin degradation, and gut barrier integrity, respectively. Collectively, these shifts support the role of the TWD in promoting a less favorable microbial environment for colon health.

Predicted microbial functions, inferred from 16S rRNA gene profiles, were modestly affected by CP supplementation and more substantially influenced by basal diet. CP intake was associated with increased predicted gene content for lysine biosynthesis, a pathway relevant to butyrate production and amino acid metabolism [70,71], as well as altered profiles of lipopolysaccharide (LPS) and monobactam biosynthesis [72,73,74]. Although the role of monobactams in this context is unclear, their presence is noteworthy given emerging links between β-lactams and anti-cancer effects [75]. These functional predictions require validation through targeted metabolomic or proteomic analyses. In contrast, the TWD had broader effects, enriching predicted pathways related to pyruvate and propionate metabolism, which are important for short-chain fatty acid production, and vitamin B6 metabolism, possibly reflecting microbial adaptation to the diet’s nutrient composition [76,77]. Together, these findings suggest that both CP supplementation and dietary background may influence microbial functional potential, although direct biochemical measurements are needed to confirm these effects.

Several limitations must be acknowledged. The AOM/DSS model primarily reflects acute rather than chronic inflammation, which limits its extrapolation to long-term IBD conditions. The study also used only healthy male mice, which reduces generalizability given the known sex-based differences in colitis susceptibility and microbiome composition. We did not include a tumor endpoint, although prior studies have shown that DAI scores and histological inflammation at day 47 correlate with later tumor outcomes [5,16,17]. Notably, an unexpected increase in DAI scores was observed in the AIN/CP group, though this change was not accompanied by differences in colon length, mortality, or pro-inflammatory gene expression. These findings suggest that if CP exerted subtle adverse effects in the AIN context, they did not manifest through canonical immune or inflammatory pathways. Alternatively, the observation may be spurious, as DAI scoring, although blinded and standardized, retains some subjectivity. Additionally, sample sizes were larger at early time points, which reduced the statistical power for recovery-phase analyses. A key limitation of this study is the absence of metabolomic or proteomic data to validate predicted microbial functions and clarify host–microbiome interactions. Such analyses were beyond the scope of this study due to the limited sample material. We also used a purified CP supplement rather than whole cocoa powder, which may differ in biological effects due to the absence of other phytochemicals [78]. Lastly, the consistent dietary intake of CPs by mice in this study does not emulate the more typical intermittent intakes of humans with a varied diet. However, IBD patients may be advised to regularly consume some dietary supplements, such as polyphenol-rich foods, food powders, or extracts, as part of their treatment plan.

## 5. Conclusions

In conclusion, this study contributes novel insights into the effects of cocoa polyphenols on colon inflammation and the gut microbiome in the context of a Western diet. Contrary to expectations, dietary supplementation with cocoa polyphenols from CocoaVia™ Cardio Health Powder did not reduce symptoms of colitis or mucosal inflammation in mice fed either a healthy diet or the TWD. However, CP supplementation led to marked changes in the fecal microbiome, including increased diversity, shifts in the Firmicutes-to-Bacteroidota ratio, and enrichment of specific rare taxa, such as the Monoglobaceae and Eggerthellaceae bacteria families. These microbiome responses were observed across all experimental phases, underscoring their robustness and potential biological relevance. Additionally, CP-fed mice exhibited improved survival during the acute colitis phase, suggesting possible protective effects under severe pathological conditions. CP supplementation did not significantly alter the expression of individual inflammatory genes but was associated with subtle shifts in pathway-level immune- and cancer-related signaling. These findings collectively emphasize the complex and context-dependent nature of cocoa polyphenols, with limited anti-inflammatory effects but consistent modulation of the gut microbiome. Further investigation is warranted to clarify the mechanisms underlying these effects, especially in chronic models of inflammation and tumorigenesis. Future research should also investigate how interactions between dietary polyphenols and the gut microbiome affect host physiology and whether these findings can be applied to human health in clinical settings.

## Data Availability

Supporting sequencing data for this manuscript are available to the public at the Utah State University Digital Commons repository, https://doi.org/10.26078/2ejq-0p45 (deposited on 18 April 2024). Available files include the .txt mapping file with sample attribute information, the .csv file with 16S rRNA sequence count data with ASV identifiers, the .csv file with taxonomy mapped to the ASV identifier, and the phylogenetic tree file. Additionally, gene expression data from NanoString nSolver analyses are available at the USU Digital Commons repository, https://doi.org/10.26078/5b52-td57 (deposited on 26 June 2025); files include the Excel .xlsx nCounter PanCancer Immune Profiling Panel Gene List, the .txt mapping file, the .txt normalized gene counts, and an Excel .xlsx file for differential expression analysis results. All other data are contained within the article or the accompanying Supplementary Materials.

## Supplementary Materials

The following supporting information can be downloaded at https://www.mdpi.com/article/10.3390/nu17152482/s1, Figure S1: Incidence of adenomas in each experimental group at the study end; Figure S2: Weekly food and energy intake over the course of the study; Figure S3: Relative spleen, liver, kidney, and cecum contents weights; Figure S4: Hierarchical clustering of all differentially expressed genes for time point, diet, or supplement; Figure S5: Heatmap depicting pathway Z scores for time point, diet, and supplement experimental factors reflecting gene expression patterns in colon mucosa tissue measured by NanoString nSolver analysis; Figure S6: Rarefaction curves by experimental group and time point; Figure S7: Taxonomic classification of mouse fecal microbiome; Figure S8: Relative abundance of select bacterial families over experimental time points and Firmicutes:Bacteroidota ratio over the time points; Figure S9: Relative abundance of select bacterial families affected by basal diet or supplement; Figure S10: Functional capacity predicted by metagenome analysis using KEGG metabolism orthology with tax4fun; Figure S11: Predicted functional capacity of the mouse fecal microbiomes; Table S1: Experimental diet formulations; Table S2: Exact value of n for study endpoints by time point, diet, and supplement groups; Table S3: Alpha diversity pairwise comparisons by experimental group within time point; Table S4: Beta diversity PERMANOVA results for pairwise comparisons by experimental group within time point; File S1: Benninghoff_project_Q1_NanoString_gene_expression_results.xlsx; File S2: Benninghoff_project_Q1_microbiome count data.xlsx; File S3: Benninghoff_project_Q1_statistical analyses results.xlsx.
