# Supplementary material for: Cocoa Polyphenols Alter the Fecal Microbiome Without Mitigating Colitis in Mice Fed Healthy or Western Basal Diets
Source: Nutrients. 2025 Jul 29;17(15):2482. doi: 10.3390/nu17152482 (PMC12348170; doi:10.3390/nu17152482)
Supplement: Supplementary file 1 [file nutrients-17-02482-s001.zip › nutrients-3753115-Supplemental_material.pdf]

nutrients

MDPI

Supplemental Material

Cocoa Polyphenols Alter the Fecal Microbiome Without Mitigating Colitis in Mice Fed Healthy or Western Basal Diets

Eliza C. Stewart [1], Mohammed F. Almatani [1,2], Marcus Hayden [1], Giovanni Rompato [1], Jeremy Case [1], Samuel Rice [1], Korry J. Hintze [3] and Abby D. Benninghoff [1,*]

* Correspondence: abby.benninghoff@usu.edu; Tel.: +01-435-797-8649

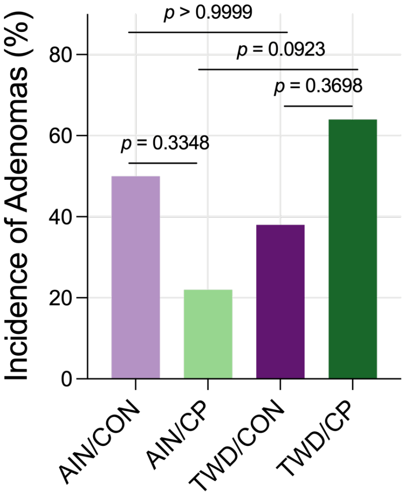

Figure S1. Incidence of adenomas in each experimental group at the end of the study. Calculated by dividing the number of mice alive within each group by the number of mice with adenomas at the study end (n = 8 to 11).

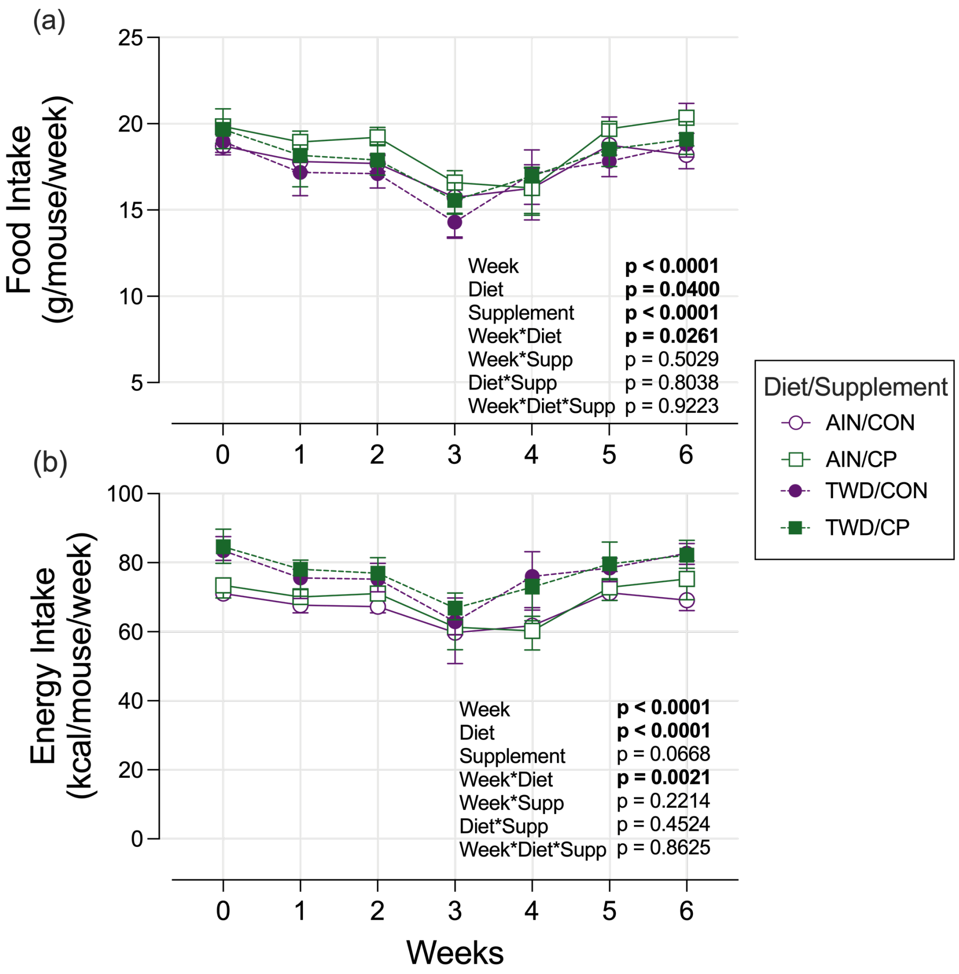

Figure S2. Weekly food and energy intake throughout the study. Reported as average food intake (**a**) and energy intake (**b**) per mouse per week. Inset tables display the statistical model's main effects and all possible interactions, as determined by the methods outlined in the Materials and Methods section.

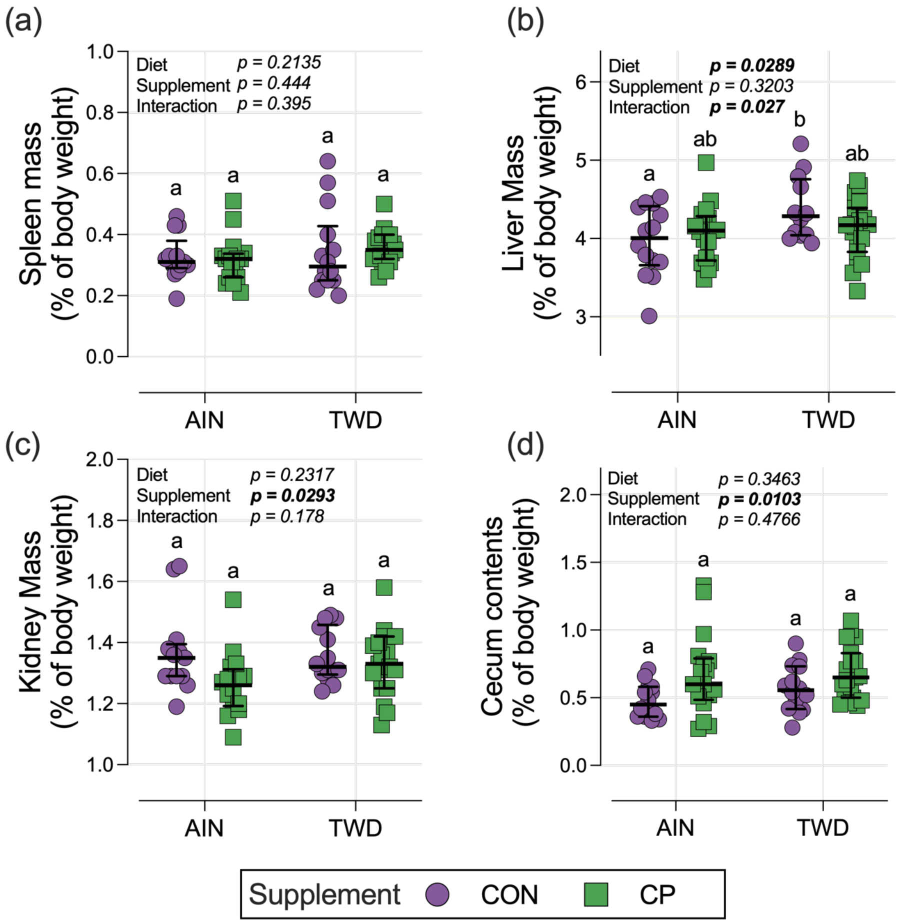

Figure S3. Relative spleen, liver, kidney, and cecum contents weights. Data for spleen (**a**), liver (**b**), kidney (**c**), and cecum contents weight (**d**) are shown as the percentage of the final body weight. Values plotted represent individual mice, shown with the median ± interquartile range (n = 14-19). Inset tables show the statistical model main effects for diet, supplement, and their interaction. Different letters in the experimental groups indicate that they are significantly different ($p < 0.05$), as outlined in the Materials and Methods.

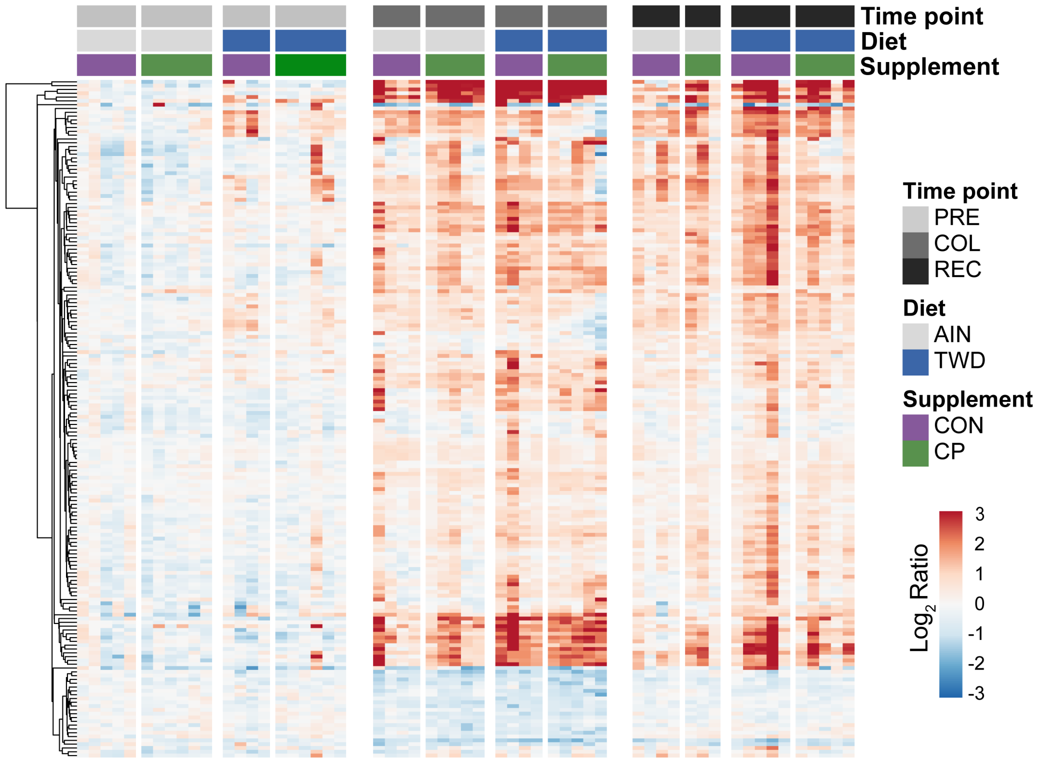

Figure S4. Hierarchical clustering of all differentially expressed genes for time point, diet, or supplement. Values are shown as the log2 ratio for each experimental factor versus the average of AIN/CON at the pre-DSS time point (PRE) (*n* = 3 to 6 for each time point/diet/supplement group).

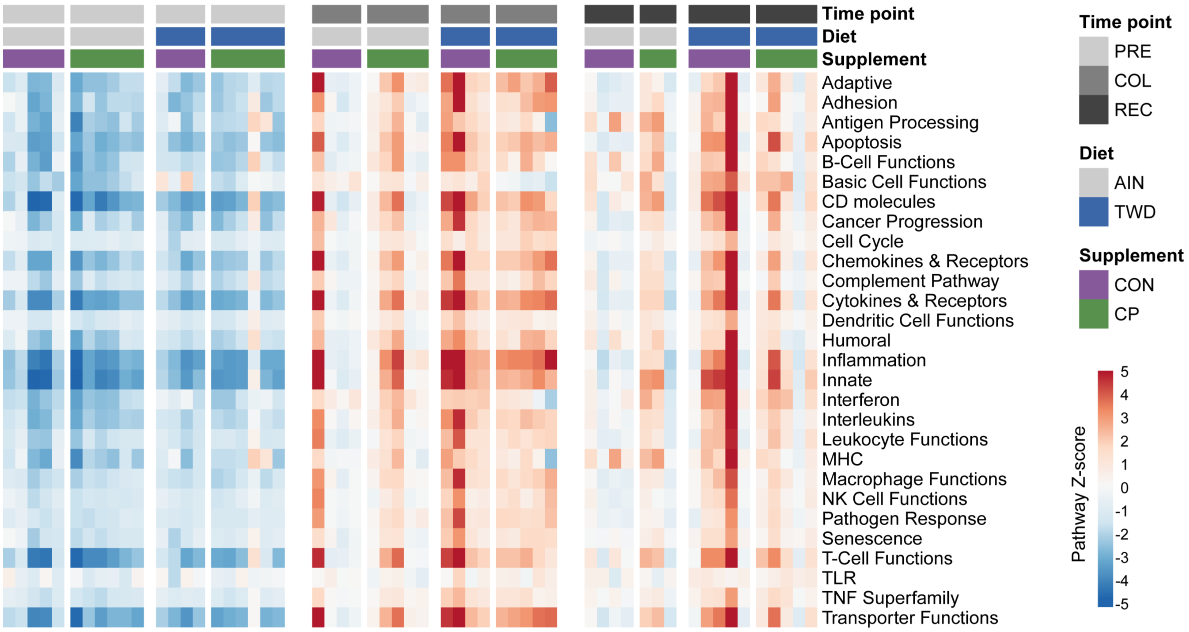

Figure S5. Heatmap depicting pathway Z scores for time point, diet, and supplement experimental factors reflecting gene expression patterns in colon mucosa tissue measured by NanoString nSolver analysis (*n* = 3 to 6 for each time point/diet/supplement group).

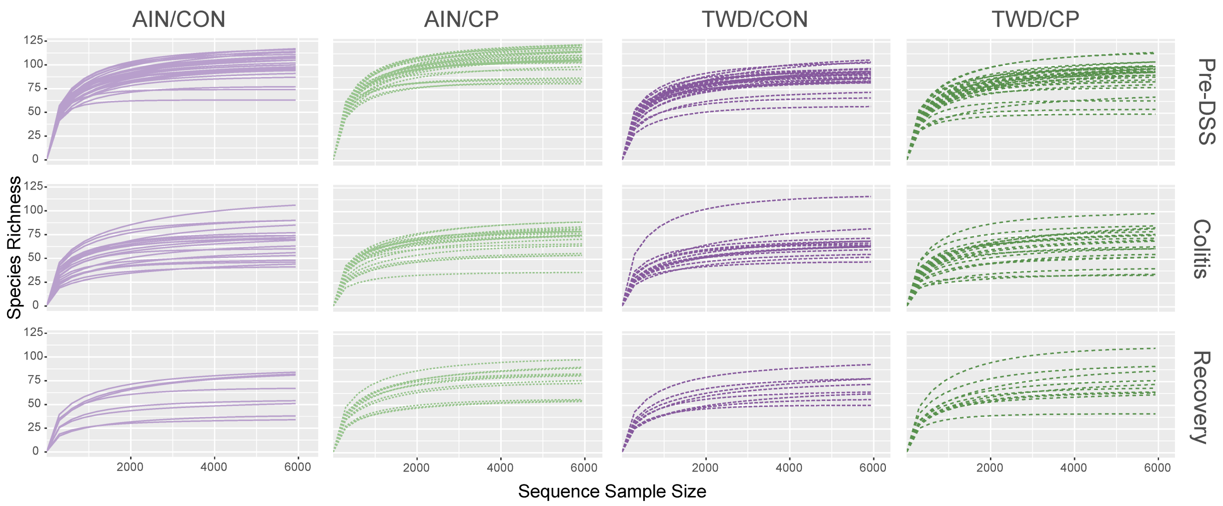

Figure S6. Rarefaction curves by experimental group and time point. Plots show species richness as a function of sequence number. For comparisons between groups, the data were rarefied to the lowest total read count for all samples, corresponding to 6,130 reads (n = 30 for pre-DSS, n = 15 to 20 for colitis, and n = 8 to 11 for recovery).

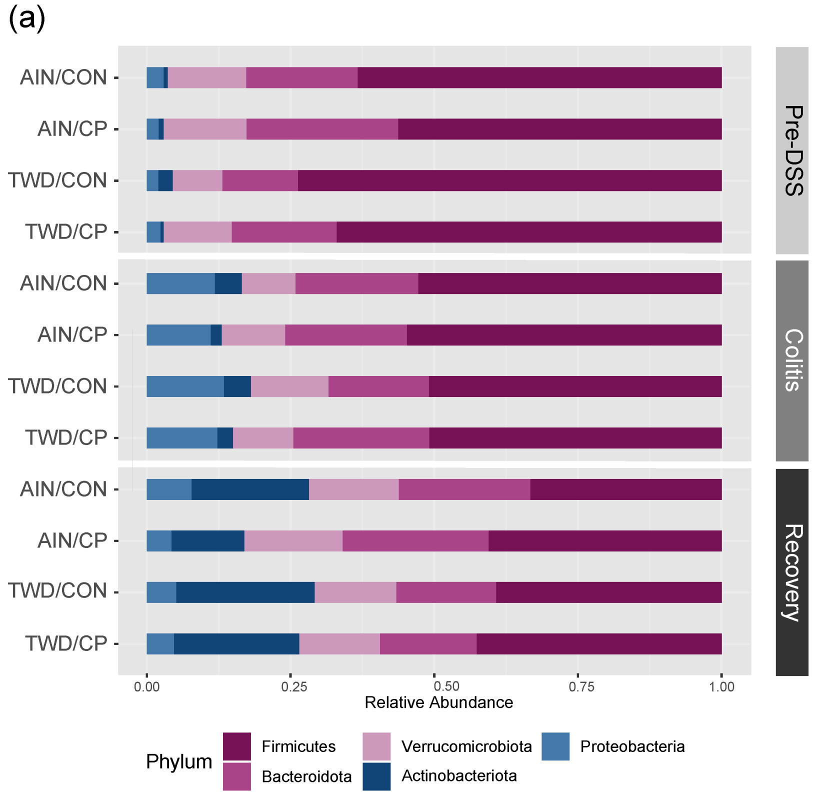

Figure S7. Taxonomic classification of mouse fecal microbiome. Data are shown as the relative normalized abundance of bacteria at the phylum level.

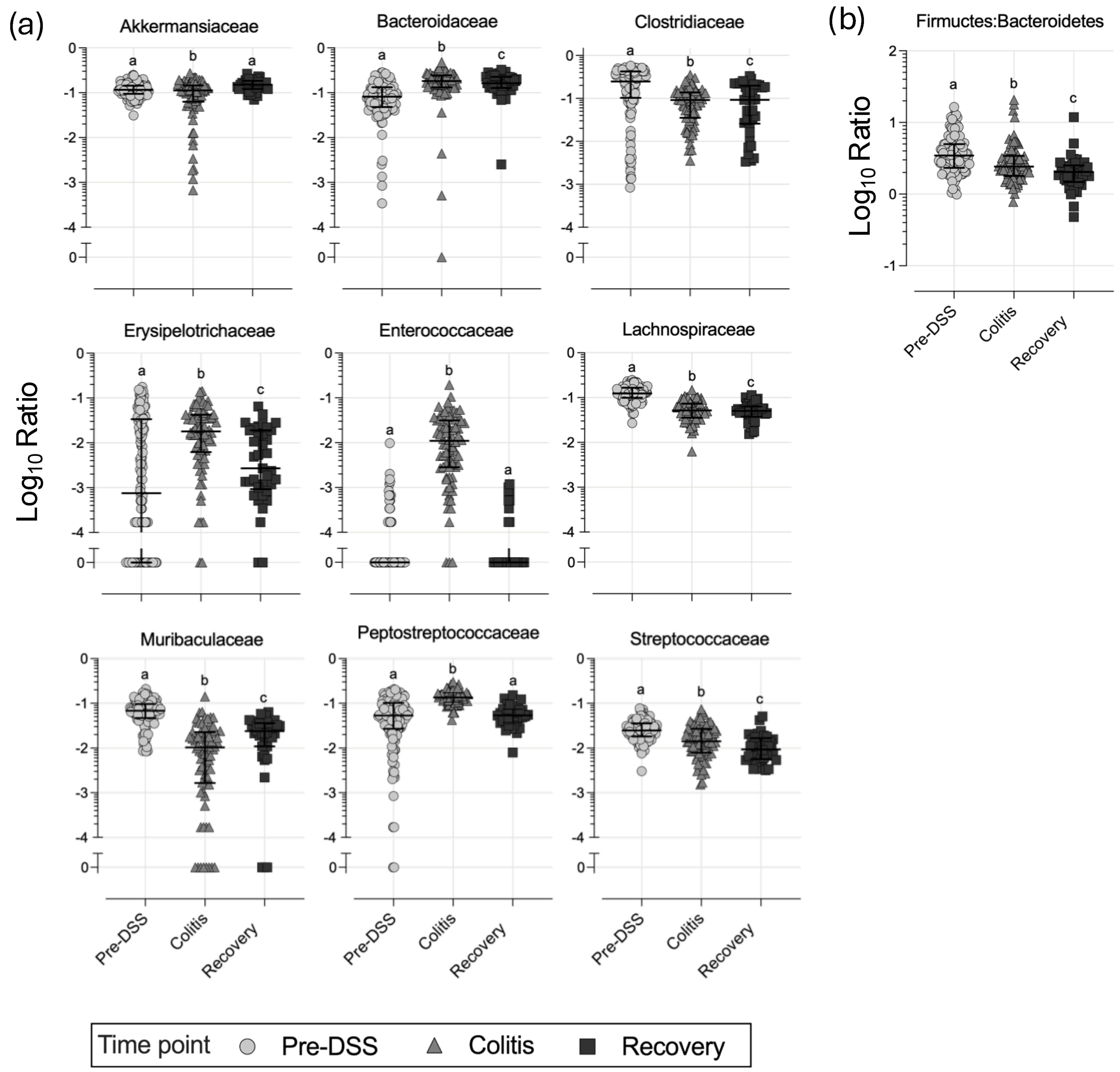

Figure S8. Relative abundance of select bacterial families over experimental time points and Firmicutes:Bacteroidota ratio over the time points. (**a**) Relative abundance of select bacteria families over experimental time points: Akkermansiaceae, Bacteroidaceae, Clostridaceae, Erysipelotrichaceae, Enterococcaceae, Lachnospiraceae, Muribaculaceae, Peptostreptococcaceae, and Streptococcaceae. (**b**) Firmicutes:Bacteroidota ratio calculated using normalized count data. Data is shown as $\log_{10}$ relative abundance of select families (**a**) and of the Firmicutes:Bacteroidota ratio (**b**) (n = 120 for pre-DSS, n = 74 for colitis; n = 37 for recovery). Individual data points represent cages and are plotted with the median ± interquartile range. Different letters indicate that the groups are significantly different ($p < 0.05$), as determined by the statistical methods described in the Materials and Methods section. See File S2 for complete MaAsLin2 and metagenomeSeq statistical analyses.

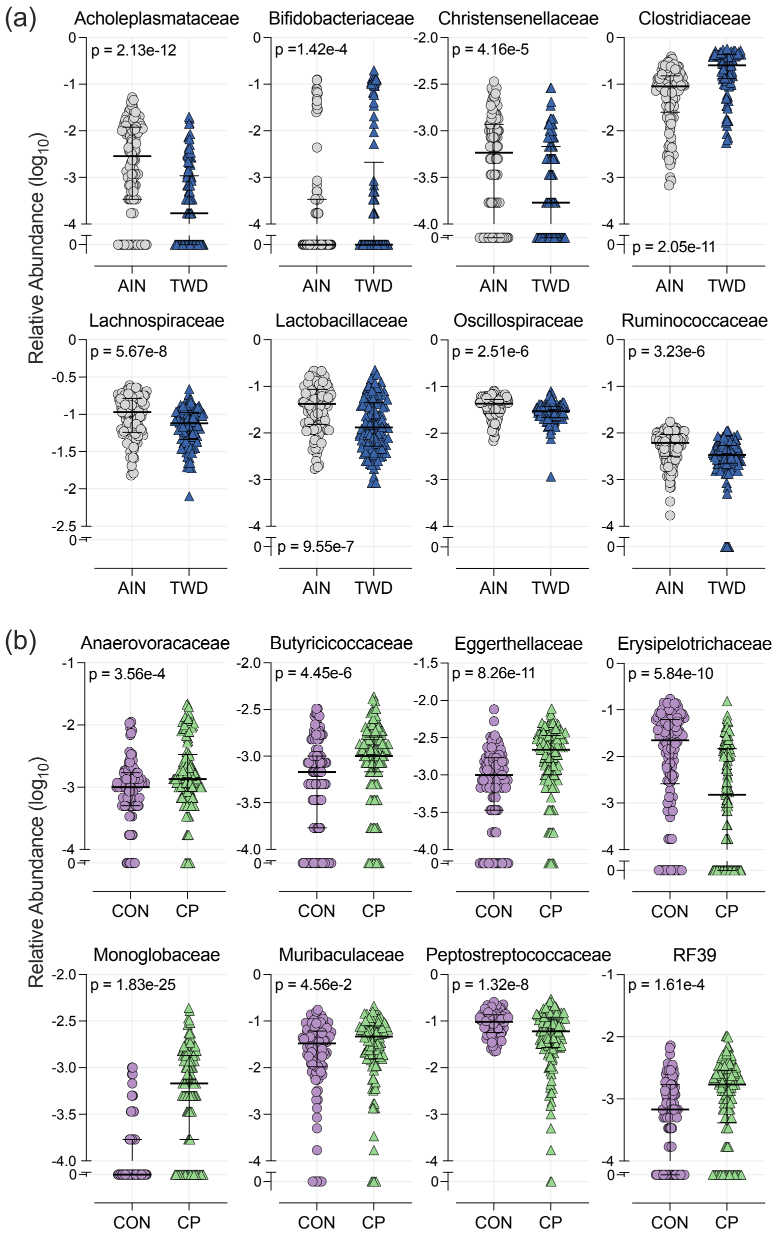

Figure S9. Relative abundance of select bacterial families affected by basal diet or supplement, irrespective of experimental time point. (**a**) Bacterial families primarily affected by basal diet: Acholeplasmataceae, Bifidobacteriaceae, Christensenellaceae, Clostridaceae, Lachnospiraceae, Lactobacillaceae, Oscillospiraceae, and Ruminococcaceae (n = 60 for pre-DSS; n = 34 for colitis; n = 16 for recovery). (**b**) Bacterial families primarily affected by CP supplementation: Anaerovoracaceae, Butyricicoccaceae, Eggertheallaceae, Erysipelotrichaceae, Monoglobaceae, Muribaculaceae, Peptostreptococcaceae, and RF39 (*n* = 60 for pre-DSS; *n* = 40 for colitis; *n* = 21 for recovery). Individual data points represent cages and are plotted on a $\log_{10}$ scale with the median ± the interquartile range. Main effects of diet (**a**) or supplement (**b**) are shown, as was calculated according to statistical methods described in Materials and Methods. See File S2 for complete MaAsLin2 and metagenomeSeq statistical analyses.

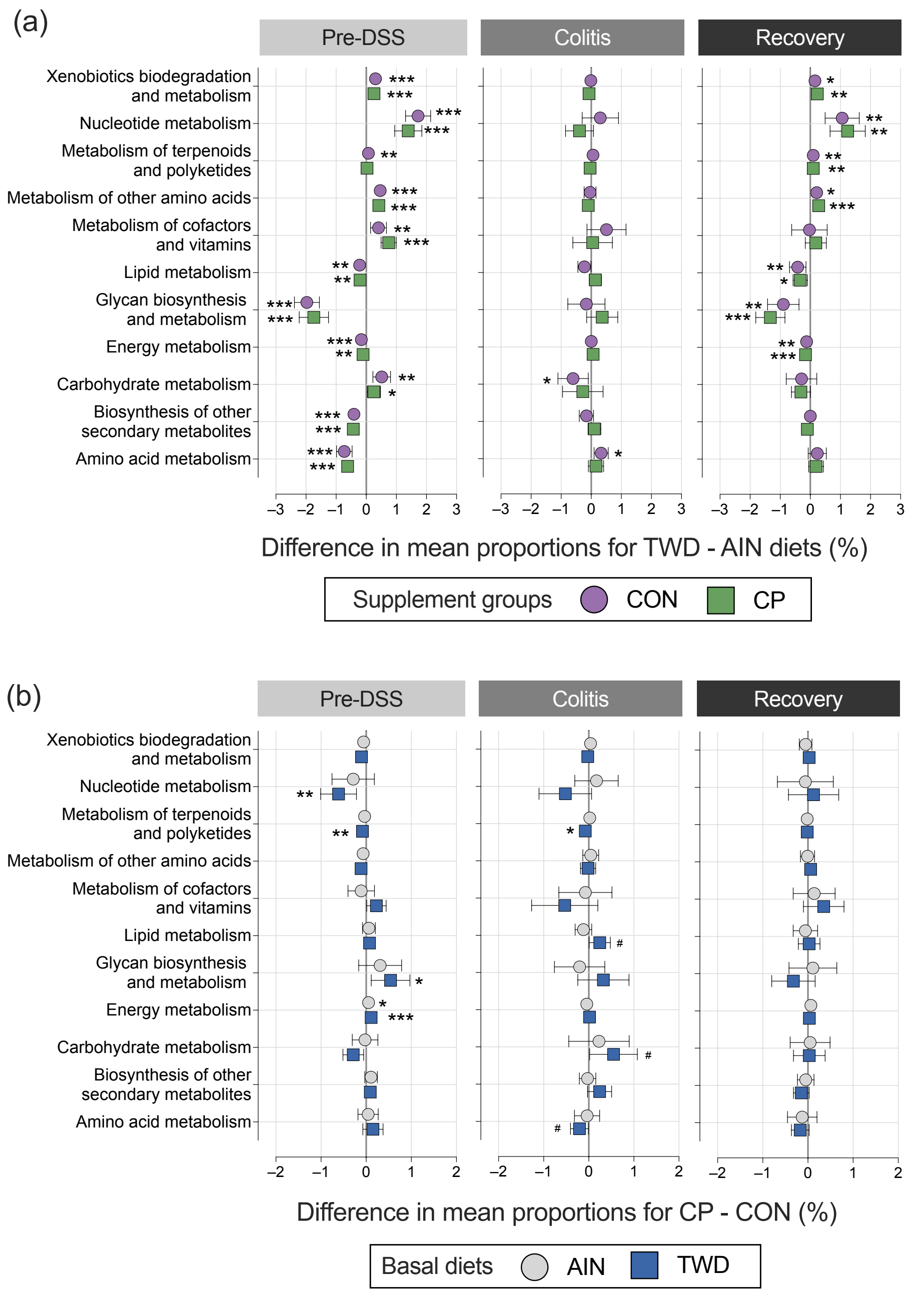

Figure S10. Functional capacity of mouse fecal microbiome predicted by metagenome analysis using KEGG metabolism orthology with tax4fun. (**a**) KEGG level 2 terms altered in the TWD basal diet compared to the AIN basal diet, plotted to show the differences between TWD and AIN in both the control and CP supplement basal diets (n = 60 for pre-DSS; n = 34 for colitis; n = 16 for recovery). (**b**) KEGG level 2 terms altered in the CP-supplemented diets compared to control diets, plotted to show comparison by supplement within the AIN and TWD diets (n = 60 for pre-DSS; n = 40 for colitis; n = 21 for recovery). Values are the differences between proportions with 95% confidence intervals. *, $p < 0.05$, **, $p < 0.01$, ***, $p < 0.001$, as determined by Student's t-test between either CP and CON or TWD and AIN with post-hoc Benjamini correction for multiple testing.

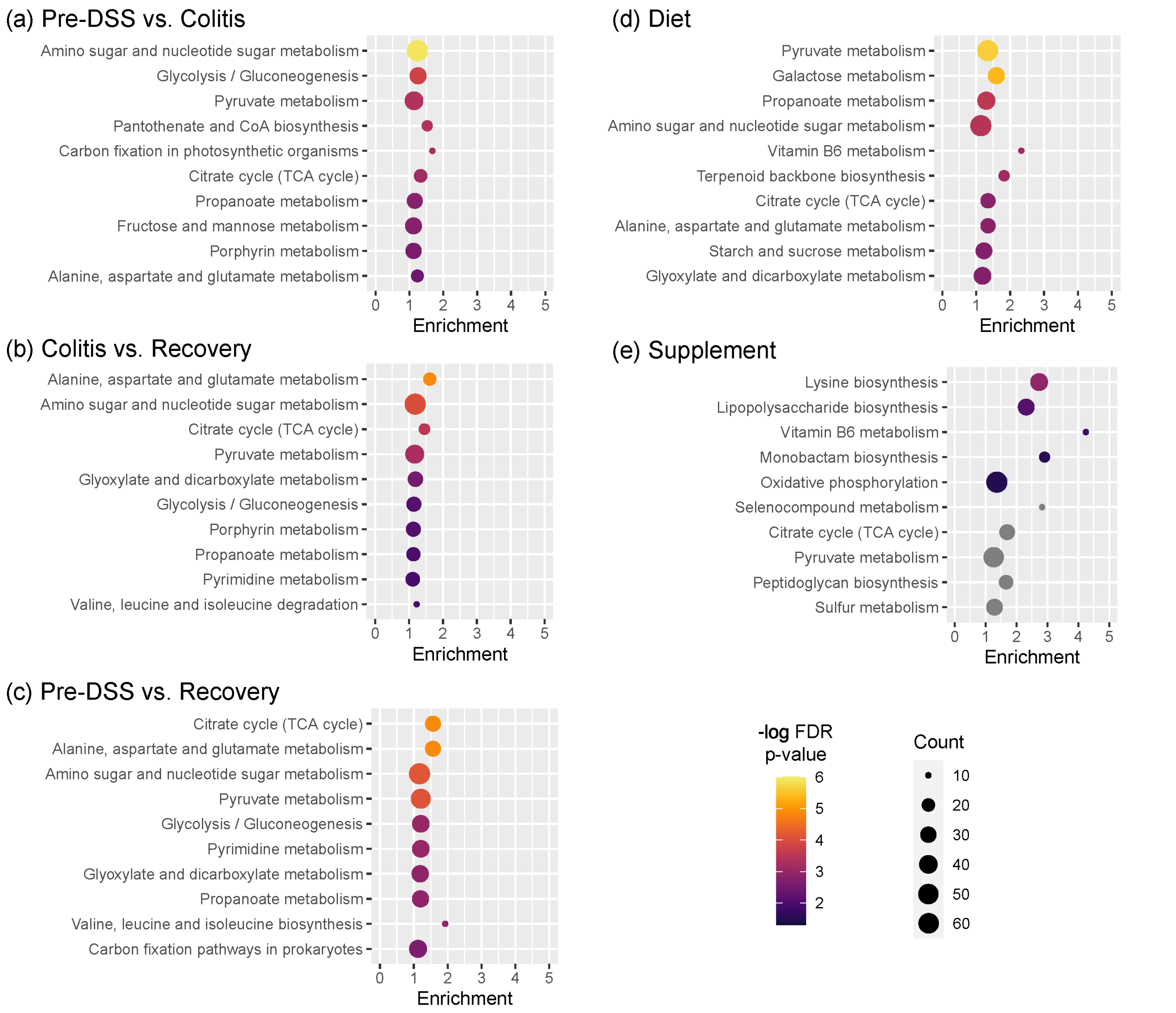

Figure S11. Predicted functional capacity of the mouse fecal microbiomes. Plots show the KEGG level 3 pathways enriched at (**a**) pre-DSS time point compared to the colitis time point, (**b**) the colitis time point compared to the recovery time point, (**c**) the pre-DSS time point compared to the recovery time point, and in (**d**) the TWD basal diet compared to the AIN basal diet and (**e**) the CP-supplemented diets compared to the CON diets. Values shown indicate the term count (number of terms associated with the metabolism pathways), the enrichment factor (frequency of terms in a pathway for a given sample compared to frequency of terms for the entire fecal metabolome), and the FDR-corrected *p*-value, as indicated in the key.

Table S1. Experimental diet formulations.

	AIN93G	AIN93G + CP	TWD	TWD + CP
Energy density (kcal/g)	3.8	3.7	4.4	4.3
Carbohydrates (g/kg diet)				
Cocoa powder		25.6		25.6
Corn Starch	397.5	388.9	230	219.9
Maltodextrin	132	132	70	70
Sucrose	100	100	261.2	261.2
Cellulose	50	38	30	18
Kcal (% of total)	*63.9%*	*63.9%*	*50.0%*	*49.8%*
Proteins (g/kg)				
Casein	200	195	190	186.6
L-cystine	3	3	2.85	2.85
Kcal (% of total)	*18.8%*	*18.8%*	*15.5%*	*15.5%*
Fats (g/kg)				
Soybean oil	70	70	31.4	31.4
Anhydrous milk fat			36.3	36.3
Olive Oil			28.0	28.0
Lard			28.0	28.0
Beef tallow			24.8	24.8
Corn oil			16.5	16.5
Cholesterol			0.4	0.4
Kcal (% of total)	*17.2%*	*17.3%*	*34.5%*	*34.7%*
Mineral mix (35 g/kg)	AIN-93G-MX (94046)	AIN-93G-MX (94046)	nTWD (110422)	nTWD (110422)
Vitamin mix (10 g/kg)	AIN-93-VX (94047)	AIN-93-VX (94047)	nTWD (110423)	nTWD (110423)

Note: Abbreviations for diets are as follows: total Western diet, TWD; Cocoa polyphenols as CocoaVia™ Cardio Health Powder[1], CP. The composition of the TWD was published previously.[2] No data on chloride, manganese, iodine, pantothenic acid, biotin, or ultra-trace minerals are available in NHANES. All diets included TBHQ antioxidant at 0.014 g/kg.

[1] CocoaVia™ Cardio Health Powder nutirition information available online. For the amount of powder added to CP diets in this study, there was an added 16 g of total carbohydrate, 12 g of dietary fiber, <4 g of protein, 4600 mg Cocoapro™ cocoa bean extract, and 2000 mg cocoa flavanols (including 320 mg of (-)-epicatechin) to the basal diets.

[2] Reference 23. Hintze, K.J.; Benninghoff, A.D.; Ward, R.E. Formulation of the total Western diet (TWD) as a basal diet for rodent cancer studies. *J. Agric. Food. Chem.* **2012**, *60*, 6736-6742. https//doi.org/10.1021/jf204509a.

Table S2. Exact value of *n* for study endpoints by time point, diet, and supplement groups.

		AIN		TWD	
Endpoint	Time point	CON	CP	CON	CP
Mortality, n (alive/dead)		40 (31/9)	40 (33/7)	40 (28/12)	40 (37/3)
Adenoma, n (none/present)		8 (4/4)	9 (2,7)	8 (3,5)	11 (7,4)
Final body weight, lean mass, fat mass		14	17	14	19
Total food and energy intake (n = cage)		8	11	8	10
DAI	Colitis	32	32	28	36
DAI	Recovery	14	17	13	19
Histopathology	Colitis	9	9	8	10
Histopathology	Recovery	8	9	8	11
Colon length	Colitis	17	16	14	18
Colon length	Recovery	14	17	14	19
Microbiome	pre-DSS	30	30	30	30
Microbiome	Colitis	19	20	15	20
Microbiome	Recovery	8	11	8	10
NanoString Gene Expression	pre-DSS	4	5	5	5
NanoString Gene Expression	Colitis	5	6	4	6
NanoString Gene Expression	Recovery	4	3	5	5

Table S3. Alpha diversity pairwise comparisons by experimental group within time point.

		Alpha diversity measure		
Timepoint	Comparison	Observed ASVs	Chao1 Index	Shannon Index
Pre-DSS	AIN/CON vs AIN/CP	0.2267	0.3143	0.1741
Pre-DSS	AIN/CON vs. TWD/CON	**0.0017**	**0.0489**	**0.0001**
Pre-DSS	TWD/CON vs. TWD/CP	0.8709	1.0000	0.0715
Pre-DSS	AIN/CP vs. TWD/CP	**0.0001**	**0.0002**	**0.0001**
Colitis	AIN/CON vs AIN/CP	0.8157	0.9300	0.7565
Colitis	AIN/CON vs. TWD/CON	0.8533	0.7641	1.0000
Colitis	TWD/CON vs. TWD/CP	0.9691	0.9405	0.9746
Colitis	AIN/CP vs. TWD/CP	0.5747	0.7084	0.4875
Recovery	AIN/CON vs AIN/CP	0.2471	0.3103	0.2311
Recovery	AIN/CON vs. TWD/CON	0.7866	0.7976	0.271
Recovery	TWD/CON vs. TWD/CP	0.9623	0.9771	0.9965
Recovery	AIN/CP vs. TWD/CP	0.9733	0.9802	0.9977

Values shown are the Tukey HSD post-hoc comparison *p*-values for each pairwise comparison between experimental diet groups within each time point following a generalized linear model analysis.

Table S4. UniFrac beta diversity PERMANOVA results for pairwise comparisons by experimental group within each time point.

		Unweighted		Weighted	
Timepoint	Comparison	R^2	*p*-value	R^2	*p*-value
Pre-DSS	AIN/CON vs AIN/CP	**0.126**	**0.001**	**0.116**	**0.001**
Pre-DSS	AIN/CON vs. TWD/CON	**0.113**	**0.001**	**0.352**	**0.001**
Pre-DSS	TWD/CON vs. TWD/CP	**0.119**	**0.001**	**0.122**	**0.001**
Pre-DSS	AIN/CP vs. TWD/CP	0.092	0.001	**0.337**	**0.001**
Colitis	AIN/CON vs AIN/CP	0.074	0.029	0.030	0.384
Colitis	AIN/CON vs. TWD/CON	0.027	0.490	0.051	0.214
Colitis	TWD/CON vs. TWD/CP	0.074	0.018	0.051	0.214
Colitis	AIN/CP vs. TWD/CP	0.056	0.038	0.059	0.384
Recovery	AIN/CON vs AIN/CP	**0.194**	**0.012**	0.074	0.216
Recovery	AIN/CON vs. TWD/CON	**0.115**	**0.041**	**0.166**	**0.009**
Recovery	TWD/CON vs. TWD/CP	**0.138**	**0.012**	**0.113**	**0.046**
Recovery	AIN/CP vs. TWD/CP	0.068	0.106	**0.279**	**0.002**

Values shown are the unweighted and weighted UniFrac beta diversity permanova multiple comparison *p*-values for each pairwise comparison between experimental diet groups within each time point.
